# Evaluating Strategies for Adaptation to Climate Change in Grapevine Production–A Systematic Review

**DOI:** 10.3389/fpls.2020.607859

**Published:** 2021-01-14

**Authors:** Audrey Naulleau, Christian Gary, Laurent Prévot, Laure Hossard

**Affiliations:** ^1^ABSys, Univ Montpellier, INRAE, CIRAD, CIHEAM-IAMM, Institut Agro, Montpellier, France; ^2^LISAH, Univ Montpellier, INRAE, IRD, Institut Agro, Montpellier, France; ^3^Innovation, Univ Montpellier, INRAE, CIRAD, Institut Agro, Montpellier, France

**Keywords:** viticulture, adaptation evaluation, drought, management practices, climate change, multi-scale, multi-criteria

## Abstract

In many areas of the world, maintaining grapevine production will require adaptation to climate change. While rigorous evaluations of adaptation strategies provide decision makers with valuable insights, those that are published often overlook major constraints, ignore local adaptive capacity, and suffer from a compartmentalization of disciplines and scales. The objective of our study was to identify current knowledge of evaluation methods and their limitations, reported in the literature. We reviewed 111 papers that evaluate adaptation strategies in the main vineyards worldwide. Evaluation approaches are analyzed through key features (e.g., climate data sources, methodology, evaluation criteria) to discuss their ability to address climate change issues, and to identify promising outcomes for climate change adaptations. We highlight the fact that combining adaptation levers in the short and long term (location, vine training, irrigation, soil, and canopy management, etc.) enables local compromises to be reached between future water availability and grapevine productivity. The main findings of the paper are three-fold: (1) the evaluation of a combination of adaptation strategies provides better solutions for adapting to climate change; (2) multi-scale studies allow local constraints and opportunities to be considered; and (3) only a small number of studies have developed multi-scale and multi-lever approaches to quantify feasibility and effectiveness of adaptation. In addition, we found that climate data sources were not systematically clearly presented, and that climate uncertainty was hardly accounted for. Moreover, only a small number of studies have assessed the economic impacts of adaptation, especially at farm scale. We conclude that the development of methodologies to evaluate adaptation strategies, considering both complementary adaptations and scales, is essential if relevant information is to be provided to the decision-makers of the wine industry.

## Introduction

Climate change adaptation is a key to the future of agriculture, a particularly vulnerable economic sector that depends heavily on weather and climatic conditions. Climate change adaptation can broadly be defined as “the set of actions and processes that societies must take to limit the negative impacts of the changes and maximize their beneficial effect” (Carter, 1996). In the case of grape growing, the potential adaptation levers are numerous, encompassing both the temporality of technical operations along the production chain—from plantation to annual crop management and winemaking, and their spatial variations due to the existing diversity of cropping systems and the close link between localization and technical adaptation (Viguie et al., 2014). It is thus interesting to understand how the research on grapevines examines management practices as well as socio-economic and cultural factors, to propose and evaluate strategies of adaptation to climate change.

It is essential that adaptation evaluation follow a comprehensive path to the understanding of climate change impacts. In the past three decades, an abundant literature—both scientific and technical—has been published on the impacts of climate change in viticulture (Mosedale et al., 2016). These impacts have been established from experiments in controlled conditions (Bindi et al., 1996; Moutinho-Pereira et al., 2009, 2010; Carvalho et al., 2016), the design of suitability maps based on bioclimatic indices (Fraga et al., 2012; Hannah et al., 2013), or crop modeling (Lobell et al., 2006; Moriondo et al., 2015). The major trends identified are: a 50% increase of biomass production in an elevated CO_2_ environment (Bindi et al., 1996); a 3 to 4 days per decade advancement of the vegetative and reproductive cycle due to higher temperatures (Caffarra and Eccel, 2011); and a higher risk of water stress impacting yield in quantity and quality (Jones et al., 2005; Schultz, 2010; Mosedale et al., 2016; Van Leeuwen and Darriet, 2016). Among these three main factors (biomass increase, cycle advancement, and water stress), the latter is the most preoccupying, as water resources are particularly vulnerable in most grape-producing areas, which are in Mediterranean climates (Medrano et al., 2015).

Although vineyard water management has been a core subject of interest for decades with regard to controlling wine quality, today climate change and the resulting water scarcity threaten yield and wine quality on an unprecedented scale (IPCC et al., 2015). This has led to studies focusing on various but complementary scales. At field scale, irrigation is one of the most effective tools to limit adverse effects of water scarcity. Medrano et al. (2015) reviewed the different irrigation management techniques designed to enhance water use efficiency (e.g., deficit irrigation, partial root-zone drying, water re-use). They also explored soil and cover crop management as a way to maximize green water use. Palliotti et al. (2014) listed the impacts of various canopy management practices to delay the advancement of ripening due to temperature and to water deficit. The selection of drought-tolerant grape and rootstock varieties (Duchêne et al., 2010; Romero et al., 2018) has also been studied. At farm scale, the study of the socio-ecological system allows different types of adaptations like wine-making innovation, yield limitation, diversification and so on to be included (Nicholas and Durham, 2012; Lereboullet et al., 2013). At regional scale, the migration of viticulture production toward higher elevation and/or higher latitude regions is also considered as an adaptation strategy (Hannah et al., 2013; Delay et al., 2015). We can thus see that there are already many opportunities for implementing a wide diversity of adaptation levers to improve the management of viticulture under future climatic conditions.

These studies are however often scattered across disciplines and have little regard for the wide diversity of winegrowing systems or for the spatial heterogeneity of water resources and climate change impacts. The focal research question is now: how does the current body of literature on the evaluation of adaptation integrate the possible trade-off between adaptations, considering both time and space? To address this question, the present study investigates the current literature to determine the ways in which adaptation levers and scales can be integrated and evaluated, and in which integrative approaches may be further developed. Recently, Santos et al. (2020) provided an updated overview of adaptation levers in viticulture on the basis of results of relevant and illustrative research. The wide-ranging scope of their review does not allow an exhaustive compilation of previous studies. In this article, we propose an exclusive compilation of adaptation evaluation only. We aim to reach both researchers and policy makers by providing a comprehensive review of the current adaptation strategies and methodologies. We explicitly focus on the adaptation to water scarcity since: (1) water resources are projected to be strongly limited by an increase of water demand and a decrease of water availability under future climatic conditions (IPCC et al., 2015); (2) water availability and water management studies require spatial and temporal variations to be considered explicitly; and (3) we assume synergies and trade-offs to exist among the numerous adaptation levers proposed at different scales (water storage/competition, water use efficiency/water needs, etc.). Here we specifically discuss how current approaches and knowledge about adaptation could be integrated into locally specific adaptation evaluation in order to provide relevant information to decision-makers.

The present paper is structured as follows. In section Methods, we present the methodology we used to select and analyze the available publications. In section Adapting viticulture to future water scarcity, we synthetize the literature on adaptation strategies, highlighting the potential synergies and trade-offs when combining levers and scales. In section Evaluating climate change adaptation in viticulture, we detail the various methodologies proposed for assessing the impact of adaptation strategies. Section Discussion discusses possible future prospects.

## Methods

### Article Selection and Analysis

In this review we applied systematic methods for document selection and inclusion, and we mixed qualitative and quantitative analyses. The literature search, conducted in June 2019 in the Clarivate Analytics' Web of Science (formerly operated by the Institute for Scientific Information) for the whole available period (1955–2019), included peer-reviewed papers, working papers, and conference presentations. The research equation was divided into three types of keywords delimited by the operand “AND” and applied on TOPIC. The first part of the equation referred to climate change “climat^*^ NEAR chang^*^ OR global^*^ NEAR warm^*^,” the second part referred to wine-growing systems “wine^*^ OR vine^*^ OR grape^*^ OR viti^*^,” and the third part referred to adaptation or water management “water^*^ OR adapt^*^.” The choice to put the operand “OR” between adaptation and water management allowed us to include studies focusing solely on water management as well as studies that considered water management practices among more general adaptive strategies. We did not specify the study scale as our objective was to compare adaptation studies at various scales, from the plant to the region. To reduce the risk of missing relevant papers, we verified that the most cited references in the collected articles were present in the search results. The initial search yielded 645 results, duplicates excluded.

**Figure 1:**
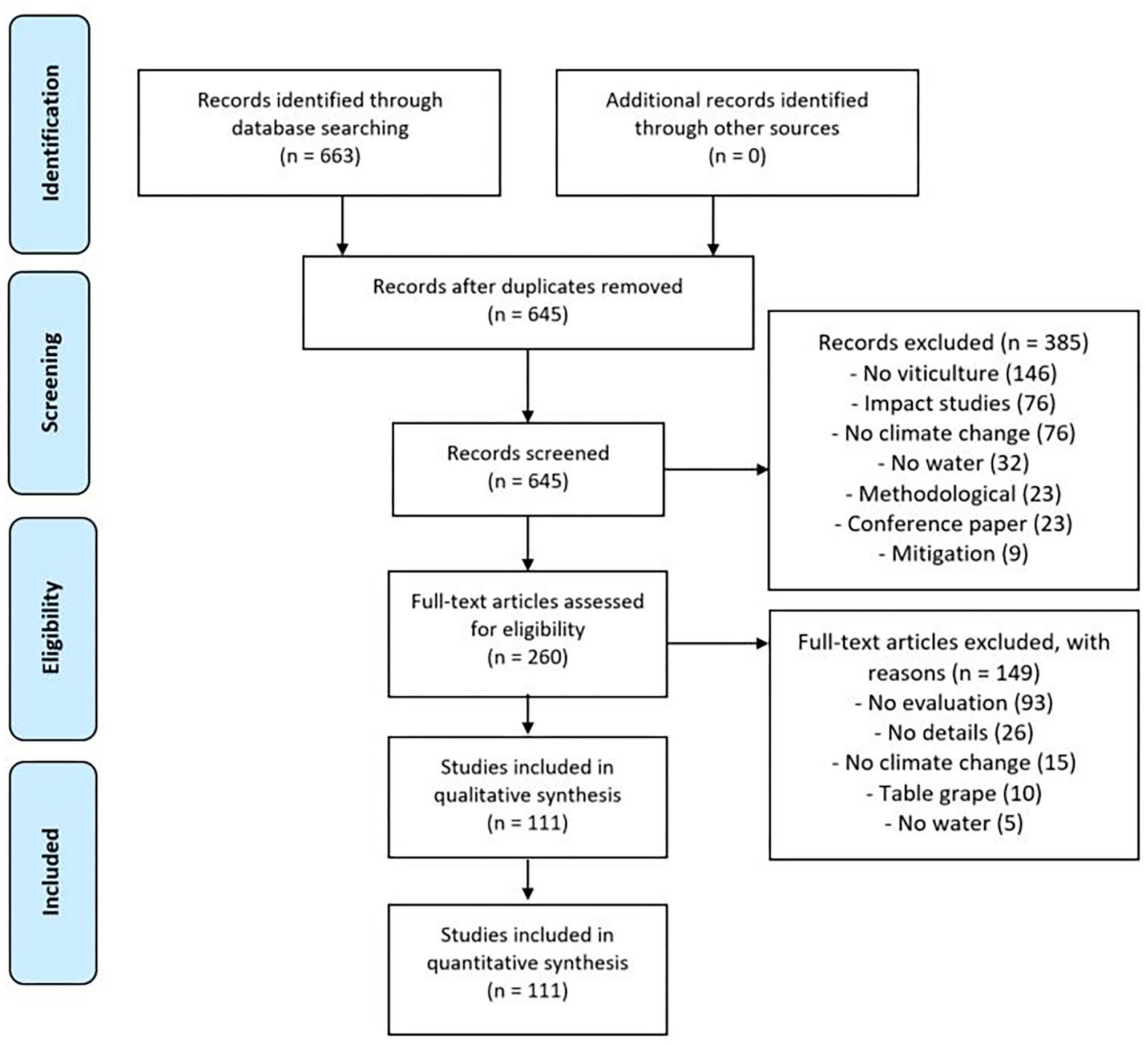
PRISMA flow diagram showing selection of papers for the final dataset (*n* = 111) (Moher et al., 2009).

Title and abstract were scanned for their relevance, articles requiring further consideration were shortlisted, and full papers were accessed. For this review, we excluded papers that did not match the following selection criteria: (1) focused on wine grape, not table grape, production; (2) construction or evaluation of adaptation strategies at the core of the study, not only in a discussion after an impact study; and (3) at least one adaptation to water scarcity was included. A total of 260 articles remained after this first selection and were read in full. Only the ones where adaptation was explicitly evaluated were included in the present study; in other words, adaptations were explicitly projected under future climatic conditions (data-based or not) and their impacts were either quantified or qualified regarding their feasibility evaluation. Our final dataset included 111 references (Figure 1; complete list in Supplementary Table 1).

First, we used the information from the Web of Science database to characterize the set of literature on adaptation for: authors, title, publication year, journal, web of science category, first author localization. We then described each of the 111 studies with categorical variables (Table 1). Analyses were performed with the R software version 3.5.1 (R Core Team, 2018), and diagrams with the “ggplot2” R package (Wickham, 2016), and the “VennDiagram” R package (Chen, 2018).

**Table 1:** List of the recorded information of the final dataset (*n* = 111).

**Variable**	**Category**
**On adaptation**	
Crop	Grapevine and others crops (including forestry)
Variety	Grape variety (e.g., Shiraz, Tempranillo)
Adaptation	Deficit irrigation, drought tolerant variety, etc. (complete list in Figure 3)
Studied area	One or several countries, worldwide (if concerns all the main viticultural areas)
**On methodology**	
Scientific approach	Experimental, modeling, expert judgement
Climate data source	Meteorological data, perceptions, climate model
Study scale	Plant, Field, Farm, Region
Evaluation criteria	Physiological, agronomical, economic, environmental (detailed list in Supplementary Table 2)

Second, we extracted two sets of variables. The first set of variables concerns the adaptations. Adaptations were first categorized according to their long- (LT) or short-term (ST) aspects. LT concerned site-specific planting choices that allow viticultural suitability to be increased, e.g., the environmental conditions in which grapevines could grow. ST concerned the flexible management that allows vine productivity to be adapted to the yearly specific climatic conditions. We then identified various adaptation categories according to their associated technical operations (e.g., fertilization, mulching, irrigation strategy, etc.). The context of the evaluation (studied area, variety, other crop considered), as well as the main impacts of the adaptation were also described. We also performed an in-depth quantitative analysis focusing on the impacts of adaptation levers on five main outputs from plant to region scale: grapevine water status, phenology, yield, berry quality (sugar content and acidity) and freshwater ecosystem (streamflow, pollution). These outputs were chosen because they were the main evaluation indicators. At least one of these outputs had been quantitatively evaluated in 43 studies, and the results of these studies were extracted. Each result [combination of an adaptation, an output and an experimental condition (year, site, simulation)] was expressed as the absolute and/or relative effects of adaptation compared to the control. We classified results according to their “positive” or “negative” effects on the outputs with regard to climate change outcomes. Positive or negative effects were not systematically similar to an “increase” or a “decrease,” depending on the considered output. For example, positive effects on phenology is a delayed occurrence of phenological stages, as climate change tends to accelerate the phenological cycle. The positive effects on water status and yield are the reduction of water stress and the increase of yield, respectively. The positive effects on berry quality are a decrease of sugar content and an increase of acidity. Negative effects on freshwater ecosystems are the reduction of streamflow and the increase of pollution. Non-significant results are classified as “neutral”. Section Adapting viticulture to future water scarcity presents the results, describing how the combination of adaptation levers at long and short term allows a better adaptation to climate change.

The second set of variables concerns the evaluation methods. We excluded the 18 review papers from our analysis (18 out of the 111 papers), as we did not consider reviewing as a way to evaluate an adaptation. Four types of information were collected: the scientific approach, the climate data source, the study scale, and the evaluation criteria. First, the scientific approach is characterized according to the three categories described in Carter (1996): experimentation, impact projections (i.e., modeling) and expert judgement. Second, the performance of an adaptation under future climatic conditions depends largely on the data used to define those conditions. Climate data sources are classified by Carter (1996) into three categories: synthetic scenarios that consist of current meteorological data adjusted systematically (e.g., +2°, −10% annual precipitation, etc.); analog scenarios based on the identification of current climatic regimes that may occur in the future (i.e., perception); and data from climate models. Third, Neethling et al. (2019) have demonstrated the importance of scales to assess expected impacts, understand uncertainty, and frame sustainable responses over space and time. Herein, we classify the articles according to the scales of the studied processes: the plant scale corresponding to the eco-physiological processes (e.g., gas exchange, photosynthesis, water status); the field scale corresponding to the agronomic processes (e.g., soil properties, yield, berry composition); the farm scale corresponding to socio-economic processes (e.g., income, cost, labor); and the region scale corresponding to agro-eco-environmental processes (e.g., streamflow, wine market, regulation). At each scale, the evaluation criteria, i.e., the specific measured, simulated, or observed outputs of the studies are listed.

### Overview of the Final Selection of Articles

**Figure 2:**
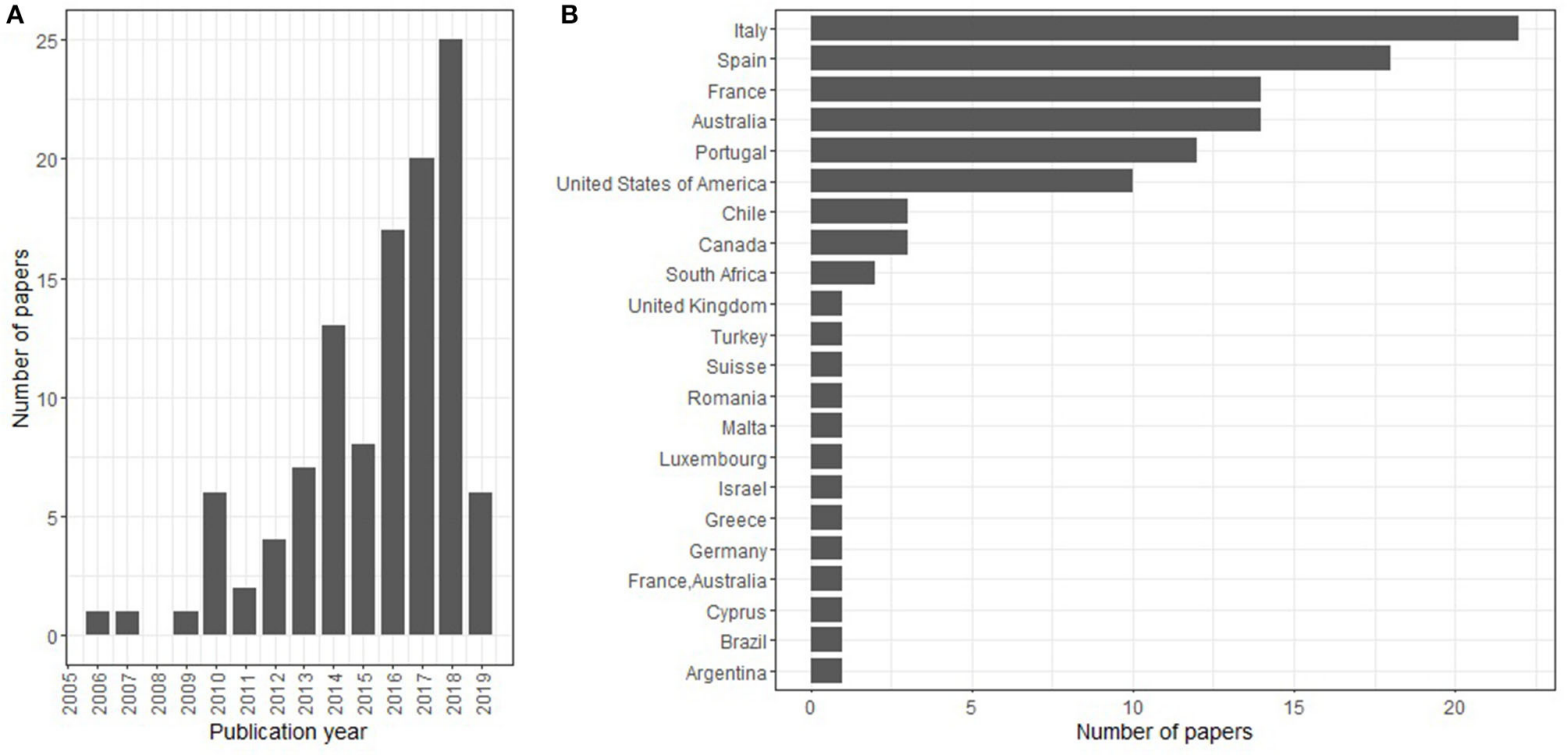
Presentation of the article pool (*n* = 111) regarding **(A)** the publication year (count stopped in June 2019) and **(B)** country of the 1st author.

In our database, the first journal article to focus on adaptation of grapevine to climate change was published in 2006 (Belliveau et al., 2006), 10 years after the first impact study of climate change in viticulture (Bindi et al., 1996). The number of papers has increased steeply since 2016 (Figure 2A).

Over the whole set of articles, authors from the Mediterranean area, i.e., Spain, Portugal, Italy, and France, accounted for 58% of the articles, followed by other major viticulture regions in the world, mainly Australia, USA, Canada, Germany, and Chile (Figure 2B). Most of the studies concern one region (87 papers) while a few others are comparative studies between countries [Australia and France (Lereboullet et al., 2013), Germany and Argentina (Uliarte et al., 2013), France, Italy, and Germany (Battaglini et al., 2009)] or a worldwide analysis (Hannah et al., 2013). The two journals that publish the most on the adaptation of viticulture to climate change are grapevine-specialized journals, namely *Australian Journal of Grape and Wine Research* and *Oeno One* (11 and 8 papers, respectively). *Agricultural Water Management, Scientia Horticulturae*, and *Regional Environmental Change* published 6 papers each.

## Adapting Viticulture To Future Water Scarcity

In the reviewed scientific literature, short-term (ST) and long-term (LT) adaptations, implemented, respectively, during the grapevine growing season and at vineyard plantation, were evaluated. Long-term adaptations concern: Site selection (LT1), consisting in the relocation of vineyards; Plant material (LT2), consisting in the implementation or creation of adapted grapevine cultivars and rootstock; Vineyard design (LT3), which implies changes in density, row orientation, training system; and Farm strategy (LT4), which includes wine-market orientation and diversification. Short-term adaptations concern: Irrigation (ST1); Soil management (ST2) concerning both soil surface (cover crop, mulching, tillage, etc.) and fertilization management; Canopy management (ST3); and Harvest and post-harvest management (ST4).

**Figure 3:**
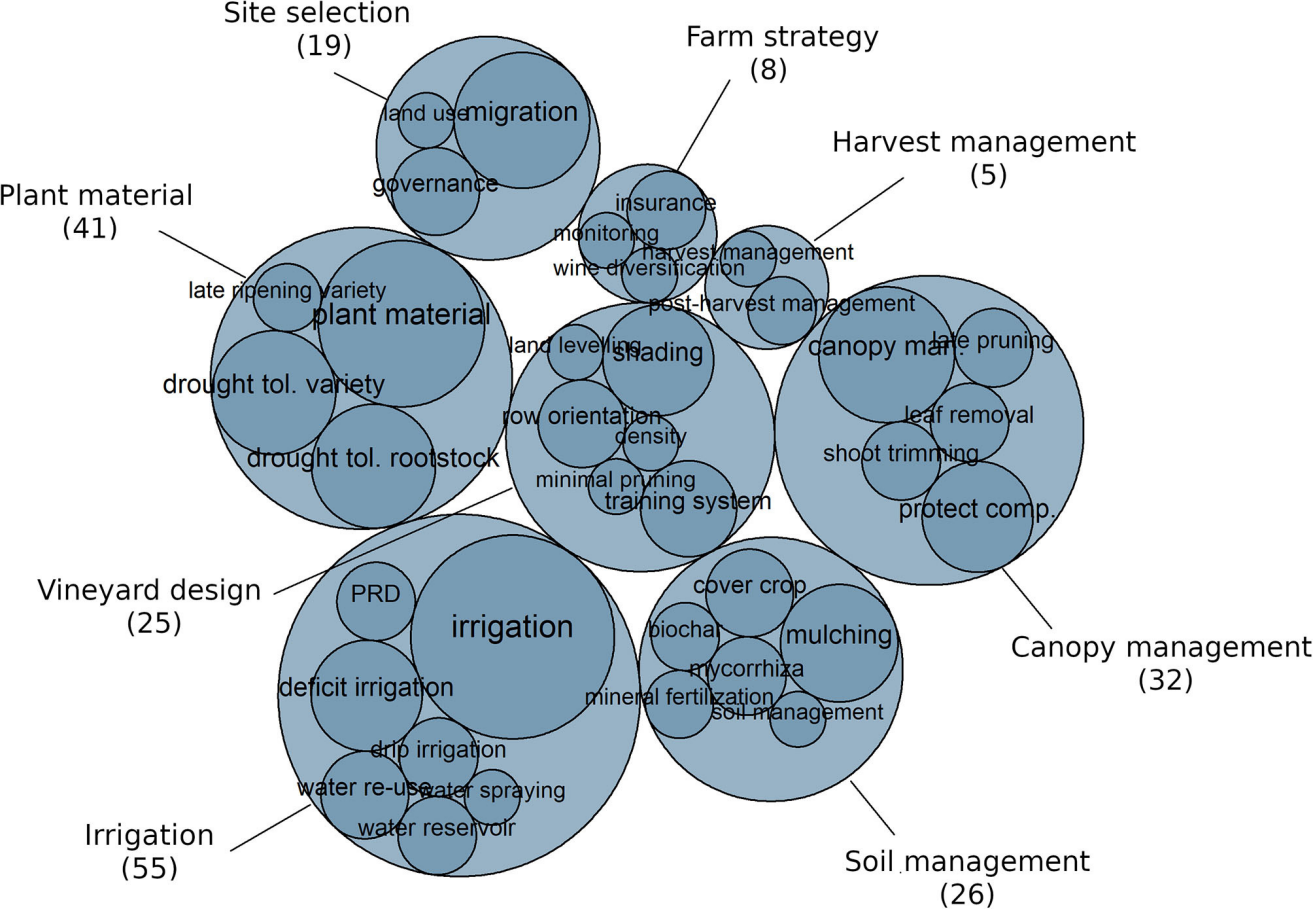
Number of studies that evaluate each adaptation lever. The size of the circles is proportional to the number of studies evaluating the levers. The number of studies appears in brackets for the main categories. One study can appear several times, as it may evaluate several adaptations.

Figure 3 represents the occurrence of each adaptation in the studied dataset. One study could be counted several times as it examined more than one adaptation. Long-term and short-term adaptations were studied almost equally with 93 occurrences of LT adaptations, and 117 occurrences for ST adaptations. We recorded 32 individual levers limiting adverse effects of climate change on water resources in the vineyard. Irrigation was the most cited adaptation (55 studies), with a wide diversity of individual levers: on irrigation strategies (deficit irrigation, partial root drying irrigation, water spraying) and water sources (water re-use, water reservoir). The plant material ranked 2nd (41 studies), and could be classified in three types of adaptation lever: drought-tolerant rootstocks; late-ripening varieties; and drought-tolerant varieties. Last of all came canopy management, soil management, vineyard design, and site selection, which received an intermediate amount of attention (19 to 32 studies), whereas farm strategy and harvest management were given significantly less attention (<10 studies each).

In the selected literature, 60% of the articles considered only one adaptation lever, 20% considered an association or comparison of two levers, and 7% considered three levers. The 17 remaining articles proposed a combination of several levers (up to 14), but in these articles, their evaluation was only qualitative.

### Combination of Long-Term Adaptations to Increase Viticultural Suitability (LT)

#### Site Selection (LT1)

Viticultural suitability has been examined mostly under future climatic conditions (Fraga et al., 2012; Hannah et al., 2013; Moriondo et al., 2013). Suitability maps provide spatial representations of bioclimatic indices for describing changes in the suitability of land for viticulture (Mosedale et al., 2016). Viticultural suitability is predicted to decrease in main wine-producing areas (25–73%), leading to a reconfiguration of vineyard locations worldwide. New areas are expected to become suitable, multiplying by a factor of 2 to 3 the wine-growing areas in Northern Europe, New Zealand and Western North America (Hannah et al., 2013). New suitable areas concern higher altitudes, as well as latitudes where annual precipitations are higher and grapevines suffer less from high temperatures.

Such alarming conclusions are however controversial within the scientific community (Van Leeuwen et al., 2013). The main limitation of these studies is the fact that they are based solely on bioclimatic indices (temperature and precipitation), without considering (1) specifically local conditions, (2) competition between various land uses, and (3) winegrower adaptive capacity to limit migration. First, local conditions [e.g., soil available water capacity (SAWC), irrigation water availability, sun exposure] are not integrated into suitability mapping studies. Second, as lands that have recently become suitable for grapevine are currently—or will become—suitable for other crops, conflicts may rise around agricultural land use and conservation policies (Hannah et al., 2013; Fuhrer et al., 2014). Third, Delay et al. (2015) demonstrated the role of stakeholders' organization for the maintenance of viticulture in unsuitable areas, with the example of the role of cooperatives, not only in conserving production levels but also in respecting the emblematic viticultural landscape structure. In case of migration, the future of abandoned vineyard areas is still an unknown. Whereas other crops could not be considered without irrigation, forestry (pine/eucalyptus) appears as a solution (Carvalho et al., 2016). Otherwise, stakeholders predict a return to shrublands with consequences on the local economy, tourism, and fire risk (García-Ruiz et al., 2011).

#### Plant Material Adapted to Site Selection (LT2 ^*^ LT1)

As the climate warms up, the phenological stages are advanced, generating concerns in the spring season when grapevines becomes more exposed to late frosts, and in summer when climatic conditions during berry ripening are less favorable (e.g., high night temperatures and water deficit). The existing phenological diversity among grapevine cultivars offers an opportunity for climate change adaptation, to limit the loss of suitable areas for grapevine (Wolkovich et al., 2017). However, suitability maps do not integrate this phenological diversity into their indicators, nor do they integrate the PDO delimitation, which restricts the implementation of specific cultivars.

The IPCC has emphasized that Mediterranean climate areas are more likely to face an increase of drought and a reduction of renewable surface water and groundwater resources in the future (IPCC et al., 2015). Accordingly, plant materiel (cultivar and rootstock) should be selected for their drought tolerance. There are many studies comparing the behaviors of various grapevine genotypes under water-restricted conditions (e.g., Tomás et al., 2014; Vaz et al., 2016). Empirical knowledge of winegrowers is reported by Lereboullet et al. (2013) in Australia: “*in 2011, many producers were starting to plant alternative Mediterranean varieties such as Grenache, Tempranillo or Mourvedre that offer a better resilience to water stress than Shiraz.”* However, the understanding of the genetic factors relevant to water stress tolerance is still limited, and quantification of yield response to water scarcity for various cultivars and in interaction with other climate variables remains difficult. An attempt to model these factors was undertaken in Tuscany. The authors found that the combination of partial uphill relocation, combined with the expansion of a drought-tolerant variety leads to a higher economic efficiency than each adaptation separately (Zhu et al., 2016). These results are however based on a major assumption that the yield of the drought-tolerant variety would not be affected by the climate change.

Duchene (2016) and Medrano et al. (2015) also highlighted the fact that rootstock-scion interaction plays a fundamental role in water use efficiency. Rootstocks have long been an unexplored field of research that is now increasingly being investigated for two reasons: its effect on root development and density, and therefore on the capacity to extract water from soil and to detect drought; and its effect on scion vigor, which influences light interception, carbon assimilation and consequently yields. Serra et al. (2014) and Zhang et al. (2016) reviewed and classified the current knowledge about the drought resistance of various rootstocks. Surprisingly, no reference to the grafting techniques (methods, period, height) have been mentioned in the selected literature, although it is a determinant of the plant's rootedness and the regulation of water flow (De Micco et al., 2018).

#### Vineyard Design (LT3)

Plantation density has a direct effect on a vineyard's water consumption. The objective is to increase drought resistance by reducing the competition between vines. Two studies based on a water balance model highlighted the potential of low-density systems as an adaptation to future water scarcity. Pieri et al. (2012) tested two planting densities (3,000 and 9,000 plants/ha) in the five main viticultural regions of France, for 3 vine cultivars that differ in terms of their phenological timing. They found that reduced planting density allows grapevine water status to be maintained within moderate limits, even under future climatic conditions. Van Leeuwen et al. (2019) went further by evaluating the economic effects of a density reduction. When density was reduced by 50%, rwater deficit was also halved, leading to higher yield at plant scale but lower yield at field scale, offset by lower costs (e.g., pruning and trellising, labor, chemicals, etc.). This demonstrated the economic viability of low-density.

Hunter et al. (2016) studied the impact of row orientation on microclimatic conditions (temperature, wind) and vine physiological status. They highlighted a lower water stress for east-west orientation, which may be induced by row orientation. In Australia, Galbreath (2014) likewise showed that east-west row orientation limited canopy temperature increase. Row orientation, as well as drainage terraces, also have an effect on water balance by reducing runoff. A study in Spain showed that drainage terraces could be expected to limit runoff volumes of between 19 and 50% at the 2050 horizon, thus favoring infiltration and limiting soil losses (Concepción Ramos, 2016).

The vine training system determines above all the light interception and bunch sun exposure, and thus the completion of berry ripening. Palliotti et al. (2014) identified adapted training systems allowing for an optimal bunch microclimate under future climatic conditions. However, it is difficult to state which training system is better adapted to drought. The only reference to drought is to a lower leaf-to-air vapor pressure deficit. It is sometimes argued that goblet pruned vines are more drought resistant (Van Leeuwen and Destrac-Irvine, 2017). We note a lack of comparison of the water use efficiency of different training systems, including traditional forms like goblet systems (Medrano et al., 2015). In Central Europe, under relatively cool climates, light pruning systems such as semi minimal pruning are promoted as an adaptation to climate change, as they present higher yields with lower alcohol degrees than vertical-shoot positioning systems (Clingeleffer, 2010; Molitor et al., 2019). However, the large water requirements of such systems would not be adapted to rainfed systems under semi-arid climate.

Shading systems are proposed as adaptation to climate change, designed to limit the effects of high temperatures and to limit evapotranspiration. Experiments with shade (e.g., natural with agroforestry systems, artificial with nets, shading panels, or photovoltaic panels) concentrate mainly on the effect of shade on the canopy temperatures. Overhead shade seems to be the most efficient way to decrease temperatures and water stress, as compared to full canopy shade, bunch shade, soil shade, and side-canopy shade (Caravia et al., 2016). More studies on the relationship between timing and duration of shading, whole-vine and specific canopy portion shading, and analysis of technical feasibility of canopy shading (i.e., suitability of training systems, mechanization of net setting and removal, cost/benefit ratio, etc.) are needed (Palliotti et al., 2014).

#### Farm Strategy in Relation to Planting Choices (LT4 ^*^ LT1 ^*^ LT2 ^*^ LT3)

Like any economic activity, wine growing needs to be viable. On the one hand, adaptation strategies should be acceptable to the producers: cost/benefit ratio, working conditions (mechanization) and labor availability. Yet no quantitative evaluations of adaptation on farm systems have been found in literature. On the other hand, adaptation strategies should also be suited to consumers' preferences. As Belliveau et al. (2006) have shown in Canada, planting new varieties can minimize market risks but increase climate risks; but it can also reduce climate risks and create marketing difficulties. These considerations are spatially and temporarily difficult to reconcile.

The long-term adaptation of viticulture to climate change is a result of current planting choices: where (low land, uphill)? What (cultivar, rootstock)? How (orientation, density, training system)? For which type of wine? While little attention seems to have been paid to the combined effect of site selection and cultivar choice, the evaluation overall of the combined effect of various practices remains poor. Moreover, the proposed long-term adaptations are rarely balanced by considering the final production objectives and economic returns that are defined and expected at farm scale.

### Combination of Short-Term Adaptations to Enhance Flexible Management (ST)

#### Combining Irrigation With Water-Saving Soil Management Practices (ST1^*^ST2)

Irrigation is part of most adaptation strategies proposed by stakeholders. Examples can be found in the South of France (Lereboullet et al., 2013; Neethling et al., 2017), Australia (Lereboullet et al., 2013; Galbreath, 2014), the USA (Nicholas and Durham, 2012), Italy (Sacchelli et al., 2016), Canada (Belliveau et al., 2006), and Spain (Alonso and Liu, 2013). However, irrigation needs, coupled with their possible satisfaction, are still not explored in socio-ecological studies. The main question remains: how much water do we need, now and in the future.

Two types of methodologies to assess future irrigation needs exist in the literature: experimental approaches and modeling approaches. Medrano et al. (2015) reviewed in detail the different irrigation strategies and their effects on physiological and agronomic parameters in field experiments. They concluded that regulated deficit irrigation (RDI) at an early or late stage is crucial for the sustainability of vineyards. They also detailed water saving practices—both agronomic techniques and genetic improvements—to increase water use efficiency under current climatic conditions. However, Bonada et al. (2018) showed that when dealing with climate change, elevated temperatures will increase water demand. Thus, the relationship between rainfall decrease and increase in irrigation needs is not straightforward. A modeling exercise by Fraga et al. (2018) highlighted that in some parts of Portugal required irrigation may exceed the reduction in precipitation, while irrigation could largely alleviate projected yield decreases. Based on the selected articles, we synthesized current and future irrigation needs according to the vineyard location, irrigation strategy and the different climate scenarios (Table 2).

**Table 2:** Irrigation strategies (FI, Full Irrigation; DI, Deficit Irrigation; PRD, Partial Root Drying) and associated water requirements in different climate scenarios (SRES, Special Reports on Emission Scenarios; RCP, Representative Climate Pathway).

**References**	**Location**	**Method**	**Irrigation strategy**	**Period**	**Climate scenario**	**Irrigation water requirement**
**Present**						
dos Santos et al. (2007)	Southern Portugal	Field experiment	FI	2002		197 mm
			50% DI	2002		99 mm
			50% PRD	2002		99 mm
Savi et al. (2018)	Italy, NE	Field experiment	Summer supplemental irrigation	2015		20–40 mm
Wenter et al. (2018)	Northern Italy	Field experiment	FI	2014–2015		72–262 mm
			DI	2014–2015		36–131 mm
Trigo-Córdoba et al. (2015), Mirás-Avalos et al. (2016)	Galicia, Spain	Field experiment	DI	2012–2014		50–79 mm
Aparicio et al. (2019)	Malta	Cost-benefit analysis	DI	Present		60 mm
Gaudin and Gary (2012)	Southern France	WaLIS model	DI	1972–2010		0–90 mm
**In combination**						
Cirigliano et al. (2017)	Central Italy	Field experiment	DI	2011–2013		125–591 mm
			DI + compost	2011–2013		125–291 mm
**Future**						
Kapur et al. (2007)	Apulia, Italy	Water balance model	FI	1970	SRES A2	320 mm
			FI	2095	SRES A2	480 mm
Fraga et al. (2018)	Portugal	STICS model	DI	2041–2070	RCP 8.5	50–250 mm
Phogat et al. (2018)	Australia	Hydrus 1D model	DI	2004–2015		350 mm
				2020–2039	RCP 8.5	250–450 mm
				2040–2059	RCP 8.5	260–460 mm
				2060–2079	RCP 8.5	240–480 mm
				2080–2099	RCP 8.5	280–500 mm

Table 2 illustrates the small number of studies that quantify irrigation needs under future climatic conditions, especially those concerning grapevine deficit irrigation in Europe—currently mostly rainfed. Future needs tend to vary widely across regions and to be double current needs in European regions. Lower increases are forecast in Australia as the current requirements are already high.

In areas where future water requirements will exceed water availability, agronomic practices may decrease irrigation needs by increasing soil water capacity and/or decreasing water losses. Canopy shade cloth and soil plastic mulch result in a 50% reduction in water use without detrimental effects on plant physiology under irrigated vineyards in Chile, through a reduction of soil evaporation or of evaporative demand (Gil et al., 2018). Transparent plastic covering (TPC) has been reported to increase water use efficiency in vineyards in Brazil, by creating higher humidity and lowering evapotranspiration as compared to open field conditions (da Silva et al., 2018). The use of organic matter as compost increases the soil water storage capacity and reduces irrigation needs (Cirigliano et al., 2017). Tomaz et al. (2017) showed that the presence of a cover crop under irrigated conditions forces the vine root system, mainly its thinner roots, to seek water in increasingly deeper soil.

Few combined adaptations under irrigated conditions have been reported, while a broader focus of attention has been given to precision scheduling and timing of irrigation supply. A wide diversity of equipment is explored: subsurface, drip, sprinkler, gravity, high-pressure system. Concerning the timing factor, tools for measuring the water status of grapevines are being developed to determine the frequency of irrigation through direct measurement of plant and fruit parameters (Scholasch and Rienth, 2019).

#### Enhancing Flexible Management Strategies in Rainfed System

##### Soil management (ST2)

Soil management is crucial to reduce water losses. As half of the water needed by grapevines is provided by rain during fall and winter in Mediterranean climates (Flexas et al., 2010), the soil has a decisive role in buffering the mismatch between water supply and demand. Two main aspects are considered in the literature: the soil structure (porosity, stoniness, deepness) impacting its available water capacity; and the soil's surface state, influencing infiltration and evapotranspiration.

In the selected literature, biochar is the most studied adaptation to improve soil structure. Biochar is a co-product of a thermochemical conversion of biomass, recognized to be a beneficial soil amendment which increases soil water retention (Amendola et al., 2017). Effects of biochar depend on its physical and structural elements, the rate of application, and the soil type (Baronti et al., 2014). While biochar is more efficient in sandy soils, the extent to which the soil's available water capacity could be improved in each production area, and whether it will be sufficient to counteract a decrease of rainfall during the vine cycle or not, is unknown. In any case, improving soil quality (organic matter and soil microbiology) will help to buffer the adverse effect of higher intra- and inter-annual climate variability.

The soil surface state largely influences the water balance (infiltration, runoff, soil evaporation). It is determined by soil type, technical operations (tillage, cover crop seedling, herbicide application) and rain intensity. Chrysargyris et al. (2018) found that no tillage compensated for the lack of irrigation, while slight tillage allowed for better rainfall infiltration. Cover crops are also promoted to enhance infiltration. The water competition induced by the transpiration of a cover crop could be limited by its partial or total destruction at the end of the rainy season. One potential adaptation measure to consider in further studies concerns mulches, that is, organic or inorganic products that may be placed on the soil surface. Mulches reduce soil compaction and retain soil moisture, regulate soil temperature and reduce evaporation. According to STICS simulations, mulches may mitigate yield decreases by 10 to 25% in Alentejo vineyards in Portugal (Fraga and Santos, 2018).

##### Canopy management (ST3)

**Figure 4:**
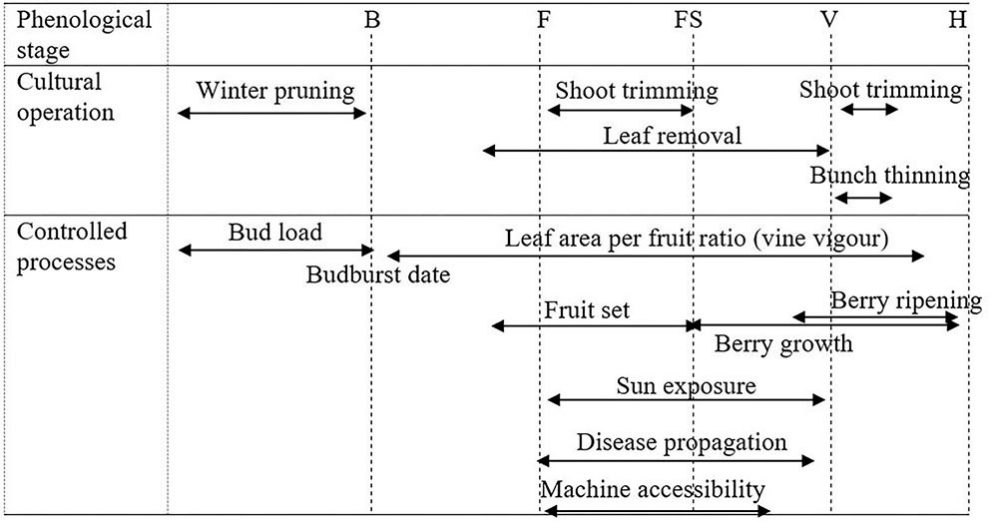
Technical operations on canopy and controlled processes (B, Budburst; F, Flowering; FS, Fruit Set; V, Veraison; H, Harvest).

Canopy management determines water consumption by controlling the leaf area index and so the transpiration rate. The diversity of operations throughout the year (winter, before flowering, after flowering, until the last days before harvest) enables a wide range of processes (Figure 4) impacted by climate change (e.g., berry ripening, sun exposure) to be controlled. The expected results from applying these techniques are closely connected to the timing and intensity of the intervention, as well as to the vine's vigor, soil fertility and environmental factors, primarily rainfall (Palliotti et al., 2014).

Leaf removal after veraison is proposed as an adaptation to climate change, as it results in a reduction of sugar accumulation rates and a postponement of the harvest date without effecting yields (Poni et al., 2018). This has been shown by tests on irrigated Sangiovese vines in Italy (Valentini et al., 2019). Results are however more mitigated under rainfed conditions (Buesa et al., 2019), which highlights the importance of environmental context on the effect of such adaptation. Although late winter pruning helps to delay ripening (Petrie et al., 2017), excessive crop load, compared to soil resources, will ultimately have negative effects on yield and grape composition and cause a delayed ripening. Yet the boundary between an adequate and an excessive crop load is not clear-cut (Palliotti et al., 2014). Şerdinescu et al. (2014) recommended the reduction of bud load only in very dry conditions.

Anti-transpirants have been used to counteract drought as their application on leaves significantly reduces water loss and heat stress (Palliotti et al., 2013). Depending on the molecules, they act in two ways: a film polymer on the leaf surfaces (e.g., kaolin); or stomatal closing compounds. They also have positive effects on the control of sugar accumulation. Their effects on plant and fruit temperatures are more contrasted, due to their effects on stomatal aperture.

##### Harvest and post-harvest management (ST4)

As the climate is changing, with higher temperatures and higher water deficits that tend to advance harvesting and affect grape composition, new harvesting management is needed. The main idea is to alter harvesting dates in accordance with temperatures (Alonso Ugaglia and Peres, 2017), but winegrowers have also envisaged other solutions. Neethling et al. (2017) identified that most adaptive responses occurred during harvest and winemaking. Harvesting with machines allows winegrowers to intervene rapidly (day and night), whereas manual harvesting systems are more restrictive. However, manual harvesting allows them to repeat the picking several times, and thus to select grape bunches that have reached their optimal maturity. Once the harvest is at the cellar, adaptations in the winemaking process are proposed. Dequin et al. (2017) recently reviewed winemaking practices adjusted to modified grape composition under climate change conditions (specific yeast strains with lower alcohol yield, membrane-based technologies to reduce the ethanol content and to increase the acidity, etc.).

### Combination of Long-Term and Short-Term Adaptation

**Figure 5:**
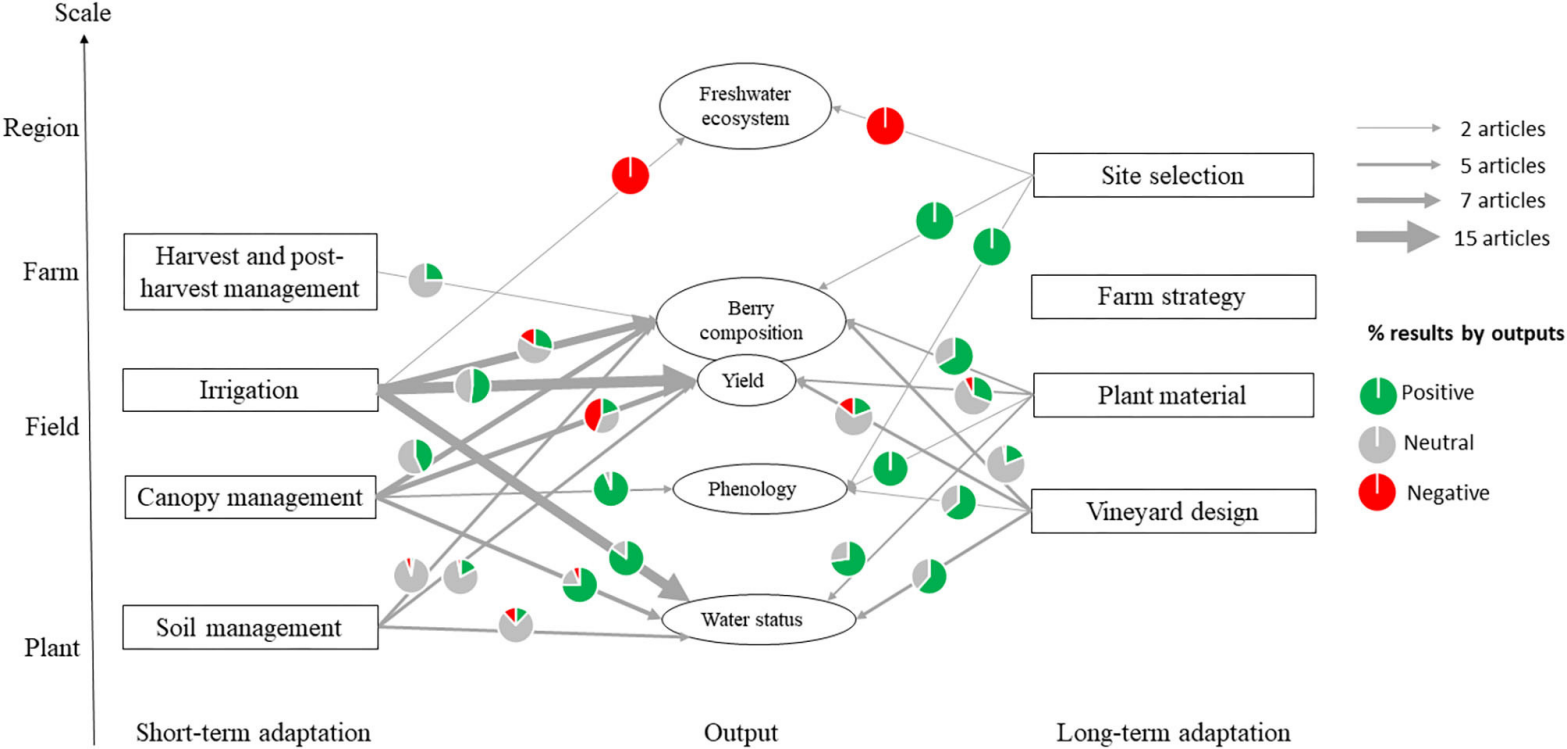
Number of articles and associated percentage of results indicating an effect of short-term and long-term adaptations on desired direction of outputs. Only significant effects (*p* < 0.05 when statistical analysis could be tested) are represented as positive or negative, and neutral means no significant effects. Positive effects on water status are those limiting water stress measured on the grapevine. Positive effects on phenology are those delaying phenological stages (budburst, veraison, or maturity). Positive effects on yield are an increase of yield. Positive effects in berry composition concern the reduction of sugar content and the increase of acidity. Negative effects on freshwater ecosystem are those reducing streamflow or increasing pollution.

The analysis of individual adaptation levers allows for potential beneficial combinations of short- and long-term adaptation to be identified. The individual effects of adaptation levers on five main outputs (water status, phenology, yield, berry composition, and freshwater ecosystem) are synthetized in Figure 5. The sources of information (papers) are detailed in Supplementary Figure 1. The majority of the impacts of adaptations concerning water status and phenology showed an alleviation of water stress and a delay of phenology for all adaptations. However, the effects on yield of these different adaptations showed contradictory results. For instance, while vineyard design and canopy management adaptations had positive effects on grapevine water status, impacts on yield are in some cases deleterious. We noticed also that the effect of irrigation on yield, which is the most studied adaptation lever, was not significant in half of the cases, thus showing that the positive effect of irrigation on yield may depend on the year and the location. The low number of articles that evaluate impacts of adaptation levers at regional scale through their effects on freshwater ecosystems is worrying, especially as all the currently available results showed negative impacts. Results on soil management adaptations were mostly not significant on grapevine outputs, while they show positive effects on soil specific outputs (data not shown).

Figure 5 allows us to identify possible tradeoffs between short-term and long-term adaptations. For example, while vineyard design adaptation (LT3) can have negative effects on yield, it could be compensated by irrigation (ST1). Likewise, the negative effect of irrigation (ST1) on berry composition could be offset by adapted plant material (LT2, e.g., drought tolerant rootstock). In the selected literature, long- and short-term adaptations were combined in the majority of studies that involved the stakeholders (Nicholas and Durham, 2012; Lereboullet et al., 2013; Neethling et al., 2017). However, quantitative evaluations of these combinations are scarce. The combined effect of variety choice and irrigation treatment has been carefully studied by Carvalho et al. (2018). The same consideration appeared recently in rootstock selection: Romero et al. (2018) demonstrated the compromise between rootstock selection and well-designed deficit irrigation strategies that allow long-term yield-quality-efficiency and returns for the grower. Vine training is also expected to influence water irrigation needs. For instance, Clingeleffer (2010) found that minimal pruning, combined with PRD irrigation, significantly increases water use efficiency compared to spur pruned and controlled irrigation treatments.

The integration of long-term considerations when evaluating short-term adaptation is crucial when dealing with economic and regulatory aspects. For instance, an analysis of technical feasibility and the economic cost of irrigation infrastructure for various localities and types of production is still lacking. Beyond irrigation needs, the irrigation decision is dependent of water resources (limited or not) and water pricing policy (Olen et al., 2016). We note the necessity to integrate the decision model into the development of irrigation-based adaptation strategies. In a trial, Trigo-Córdoba et al. (2015) estimated that irrigation is not economically viable under the current conditions of Galicia vineyards, considering both yield and quality, even though there is a physiological need for irrigation. In the vineyard of the “Old World,” irrigation was recently authorized (since 1996 in Portugal and since 2006 in France) and is still limited in “Protected Designation of Origin” areas, which can considerably change the feasibility of irrigation-based adaptations.

In addition to the economic aspect, some authors have looked at whether irrigation is an environmentally sustainable trend in semi-arid areas. One aspect is ecosystem protection, which was examined by Grantham et al. (2010), who evaluated the impact of small storage ponds on streamflow. They showed that strategic placement of storage ponds could reduce summer water withdrawals, thus protecting environmental flow. However, this could have an impact on winter flow. The development of high water-use efficiency systems in areas previously not irrigated still results in an increase of total water use. The second aspect deals with salinity problems, which appeared first in countries like Israel and Australia (Phogat et al., 2018). Model simulations indicate a steep increase of salinity in the root zone as rainfall-induced salt leaching declined significantly with climate change. The simulated seasonal average salinity increased three to four times compared to the baseline (Phogat et al., 2018). Adaptation strategies should include salinity tolerant rootstock, or the use of desalinated water (Aparicio et al., 2019).

## Evaluating Climate Change Adaptation in Viticulture

### Characterization of Climate Change

Characterizing future climatic conditions is the first step to evaluate an adaptation strategy, as its effectiveness will depend on local climatic conditions. Climate is a complex phenomenon involving many variables on different spatial and temporal scales. The ability to forecast climatic conditions is limited by the uncertainty about future greenhouse gas emissions and by the scientific uncertainty of their effects on climate and crops. The effects of combined climatic factors (e.g., higher CO_2_ concentration with higher water deficit) need to be considered simultaneously. In addition, spatial resolution of climate information is crucial to predict local phenomena. The performance of an adaptation under future climatic conditions therefore depends largely on the data used to define future climatic conditions.

First, future climatic conditions are described as a systematic adjustment of present meteorological data (e.g., a temperature increase of 2°C, a 50% reduction of rainfall, etc.) in 35 studies in the article pool (Table 3). Climatic conditions can be directly measured under controlled conditions. Controlled experiments evaluate the combined effects of different climatic changes, as for example the effect of water stress induced by deficit irrigation under elevated temperatures created with open top chambers (Torres et al., 2017; Bonada et al., 2018). Experiments that reproduce elevated CO_2_ conditions remain rare and limited to climate change impact studies without the introduction of an adaptation (Bindi et al., 1996; Wohlfahrt et al., 2018).

**Table 3:** Number of studies that describe future climate according to meteorological data, climate model, or stakeholders' perception.

**Climate data sources**	**Number of articles**
Unspecified	27
Meteorological Data	35
Climate modeling	16
Perception	12
Meteorological + Perception	1
Climate modeling + Perception	2

*The 18 review articles are excluded*.

Second, climate models (16 studies, Table 3) provide long and complete series of daily variations of a wide range of meteorological variables (CO_2_, temperature, rainfall, etc.) for the past and next centuries. Ongoing advances in modeling allow global climate models (GCM's) to be downscaled to regional climate models (RCM's) and their microclimatic versions (Quénol et al., 2017). However, the use of several models is still recommended to account for their intrinsic uncertainties. While changes in average daily climate parameters such as temperature or rainfall could be described by climate models, this approach still hardly represents extreme weather events and sub-daily variations (e.g., extreme temperature, heavy rains).

Third, stakeholders' perceptions and experiences are the basis of 12 studies (Table 3) to describe future climatic conditions. Future climatic conditions are the result of elicitation exercises which may be individual (Nicholas and Durham, 2012; Neethling et al., 2017; Bardsley et al., 2018) or collective (Lereboullet et al., 2013; Galbreath, 2014). They have the advantage of being locally adapted and of representing extreme events with their consequences. However, climate change may tend to be underestimated as the disruptive climatic conditions and new combinations of stresses, which may go far beyond local experiences, are hard to explore.

In a quarter of the selected studies, the climate evolution was not clearly specified (Table 3). The use of climate projection datasets is the only credible tool available for simulating the physical processes that determine climate change (Carter, 1996). However, it does not necessarily represent all the events proposed by stakeholders, notably those highlighted by Bardsley et al. (2018): extreme events (heat waves and heavy storms) and changes in natural resources (rainfall during the growing season and volumes of groundwater recharge). Data sources for future climatic conditions are poorly hybridized, despite the complementarity they offer. In our dataset, a single study coupled past evolution of meteorological data with winegrowers' perceptions (Lereboullet et al., 2013). Similarly, only two studies (Table 3) combined climate modeling at local scale with stakeholders' perceptions (Sacchelli et al., 2017; Tissot et al., 2017).

### Approaches to Evaluate Adaptation Effects

**Figure 6:**
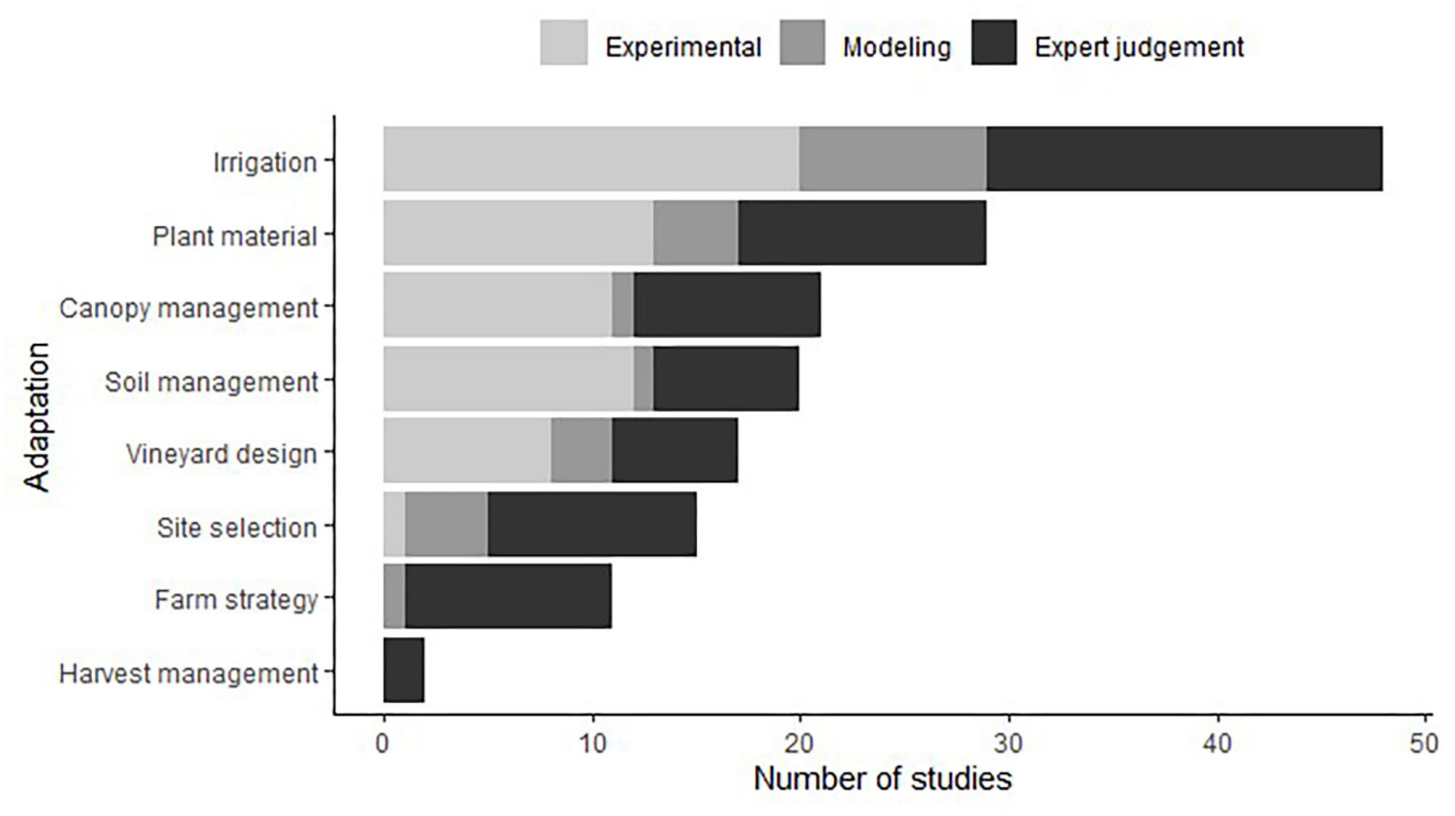
Number of studies (review articles excluded) that evaluate adaptation according to the implemented approach (experimentation, modeling, expert judgement). One study can appear several times, as it may evaluate several adaptations. We have not found any studies that combine two methods.

Figure 6 illustrates the approaches used in our pool of articles to evaluate each category of adaptation. The number of studies that employed experimental and expert assessments is similar (34 and 35, respectively), whereas modeling approaches concern 21 studies. We did not find studies that used a combination of two approaches to evaluate an adaptation. It is noteworthy that all the adaptations were evaluated by experts, and that a few of them were also evaluated by modeling or experimental approaches (harvest management, farm strategy). By contrast, some specific adaptations (not detailed in the figure), such as biochar application and protective compound, were studied through experimentation only, and have never been reported by other types of study.

Experimental approaches have been widely used to understand vines' responses to changes in climatic conditions. Controlled conditions allow for the study of processes when one or several environmental factors are changed: CO_2_ enrichment (Bindi et al., 1996), experimental drought (Medrano et al., 2003; Şerdinescu et al., 2014; Vaz et al., 2016; Cirigliano et al., 2017; Chrysargyris et al., 2018), or elevated temperatures (Bonada et al., 2018). The conditions of experimentation largely differ: from greenhouse conditions with fruit-bearing cutting under totally controlled conditions (Torres et al., 2017), to less controlled field experiments. Even if combinations of climatic factors are starting to be studied at plant scale, it is clearly difficult to extrapolate results at larger scales (e.g., field, region). The interactions between soil, climate, and cultural practices are difficult to identify fully. Moreover, the conditions of field experiments may not accurately reflect the overall production system constraints (vine age, cash flow, labor availability, water availability, etc.).

Unlike the experimental approach, which produces knowledge about the impact of environmental variables on a few processes only, models try to integrate that knowledge in order to predict the combined effects of climate change on the whole plant. Several approaches have been developed: empirical models, process-based models, suitability mapping, agent-based models, etc. We will not detail all existing models as they have been amply illustrated in a recent review by Moriondo et al. (2015). The aim of this section is to describe the types of models that are mostly used and how they are applied to evaluate combined adaptations. Suitability mapping has been used in four studies of our dataset, mainly to evaluate site selection and irrigation adaptations (Hannah et al., 2013; Fuhrer et al., 2014; Teixeira et al., 2014; Resco et al., 2016). Empirical models have been used in one study to evaluate the effect of various cases of irrigation management under future climate change (Teixeira et al., 2014; Olen et al., 2016). However, empirical models show their limits when evaluating an adaptation under alternative management conditions and future climatic conditions on which experiments have not yet been run. Process-based models have also been used in 8 studies to evaluate adaptations dealing with irrigation (Grantham et al., 2010; Pieri et al., 2012; Fraga et al., 2018; Phogat et al., 2018), plant material (Pieri et al., 2012; Zhu et al., 2016), planting density (Van Leeuwen et al., 2019), site selection (Carvalho-Santos et al., 2016; Zhu et al., 2016) and mulching (Fraga and Santos, 2018). The development of models is limited by controversial effects of climate change on various processes, such as the effect of CO_2_ on stomatal conductance. In addition, they poorly represent the perennial aspect of grapevines, as the multi-year succession of stresses and the age of the vine are not considered.

The first actors of adaptation are the decisions makers (policy makers and winegrowers). Yet both experimental and modeling approaches have rapidly derived into “top-down” approaches, moving from global climate model scenarios to impact studies, and then to assessments of adaptation. Hence, methodologies based on expert judgement have been implemented, resulting in qualitative or semi-quantitative results. Quantitative studies are mostly based on the dissemination of questionnaires in the vine industry. They allow for comparison of climate change adaptation under various macro-climatic conditions (Battaglini et al., 2009), and identify trade-offs, opportunities, and hurdles. Qualitative studies are more diverse (socio ecological studies, regional risk assessments, semi-structured interviews, etc.). These approaches deal with multiple scales and multiple adaptations, and consider a multitude of external factors. Two studies employed agent-based models to develop decision support systems that combine dynamic models with expert judgements (Delay et al., 2015; Tissot et al., 2017). These agent-based models are considered to be particularly appropriate tools for simulating complex interactions between ecological and social components (Tissot et al., 2017).

### Evaluation Scales and Criteria

**Figure 7:**
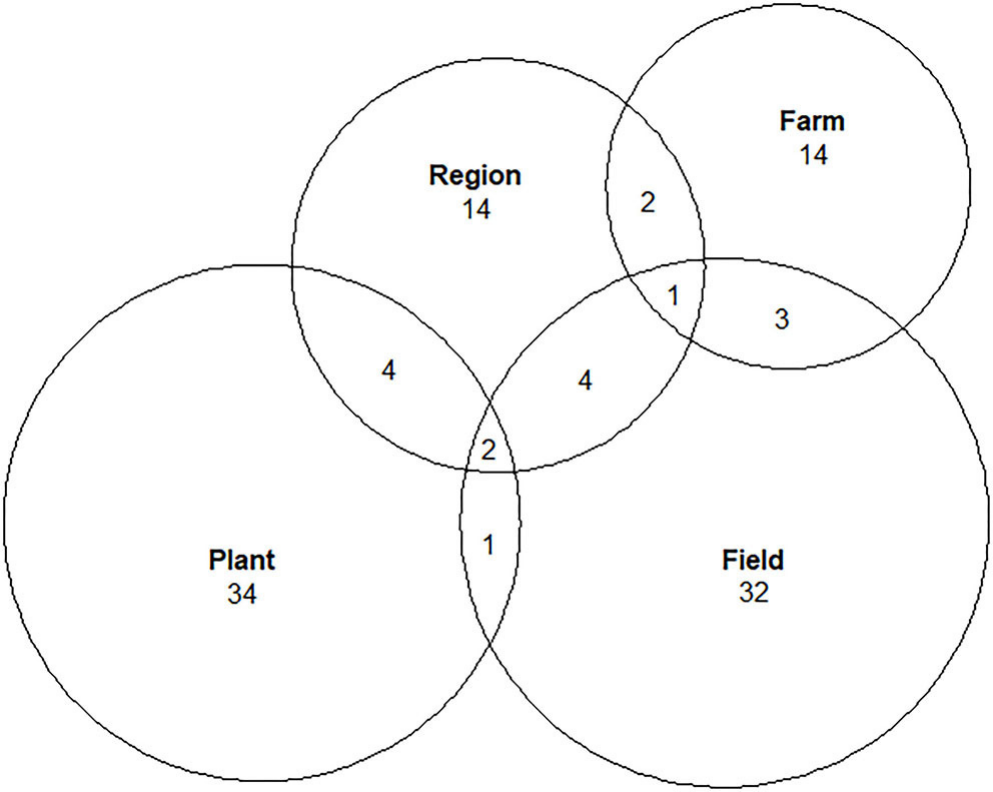
Number of studies that address one or more spatial scales in the pool of articles (*n* = 111).

Among the selected articles, 33 studied plant scale, 32 studied field scale, 14 studied regional scale, and 14 studied farm scale (Figure 7). Most of them focused on one scale, and only 17 studies considered two or more scales simultaneously. It is noteworthy that 5 out of those 17 studies applied the expert judgement methodology (Battaglini et al., 2009; Lereboullet et al., 2013; Neethling et al., 2017; Tissot et al., 2017; Bardsley et al., 2018). Upscaling can be seen as “abrupt” in some studies (e.g., Hannah et al., 2013, Fraga et al., 2018). For example, moving from field to regional scale without considering the intermediary farm scale, implies that the constraints and opportunities of the farming system are not considered (farm delimitation, wine-making processes and sales, labor availability, etc.). In the same way, the scaling-up between plant and regional scale overlooks agronomic practices than can influence the performance of an adaptation.

**Figure 8:**
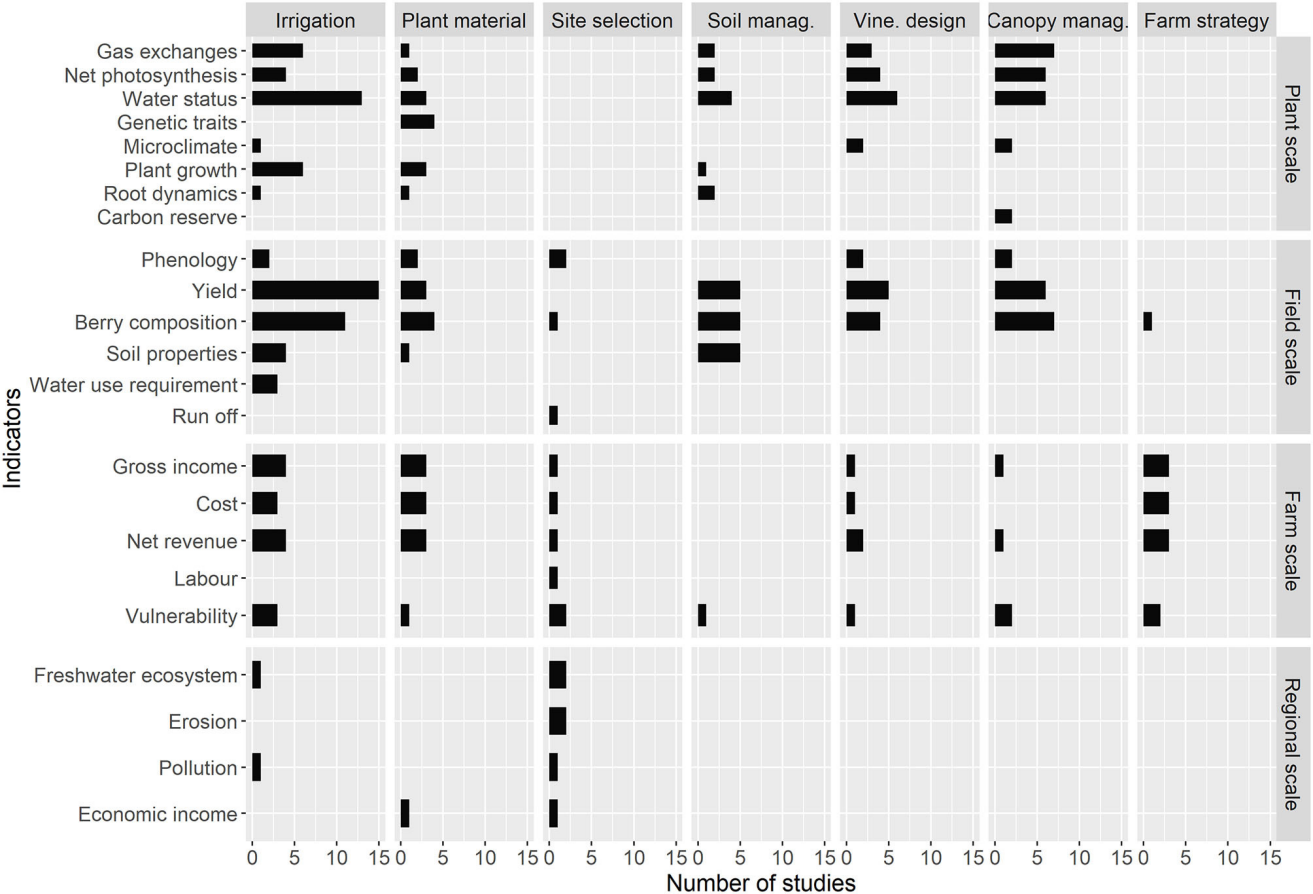
Number of studies that evaluate adaptation according to their evaluation indicators, from plant scale to field, farm, and regional scale (from top to bottom). Only quantitative evaluations of adaptation are included (60 studies). Detailed table and references in Supplementary Table 2.

Figure 8 indicates the number of studies that quantified one or several indicators for each adaptation. We see a large number of indicators at plant and field scales, whereas farm and regional scales are studied less. Yield, berry composition and water status were the most studied indicators (31, 30, and 31 studies, respectively). Seven studies addressed regional scale in a quantitative way (reference in Suppl. Mat) and concern mainly irrigation and site selection adaptation. The lack of multi-year processes at plant and field scale is noteworthy (for example, the mortality rate).

## Discussion

### Identifying the Site-Specific Trade-Off Between Adaptations

Most potential adaptations to water scarcity under future climate change have been evaluated individually. Our review suggests that the few existing studies dealing with combinations of adaptations help in identifying several compromises between these adaptations: the reduction of irrigation requirement through water-saving practices (Cirigliano et al., 2017; Chrysargyris et al., 2018; Gil et al., 2018; Romero et al., 2018; Torres et al., 2018); the benefits of cover crops despite water competition (Tomaz et al., 2017); the conservation of vineyard areas thanks to cultivar changes and new governance modalities (Galbreath, 2014; Delay et al., 2015; Zhu et al., 2016; Morales-Castilla et al., 2020); and the role of socio-economic conditions in promoting or regulating adaptations (Olen et al., 2016; Georgopoulou et al., 2017). It is noteworthy that since the systematic review ended, new developments have been published: Buesa et al. (2020) confirm the positive effects of east-west row orientation on yields; Morales-Castilla et al. (2020) quantified the reduction of suitable area lost thanks to late-ripening cultivar (from 56 to 24%); Phogat et al. (2020) went further in the estimation of future irrigation water requirement and demonstrate the importance of reducing evaporation loss; while López-Urrea et al. (2020) quantified the effect of organic and plastic mulch on evaporation. Our findings are in accordance with the climate adaptation wedges concept developed by Diffenbaugh et al. (2011). These authors illustrate the benefits of adding two adaptation strategies in limiting adverse effects of climate change in a changing context (population, development, etc.). For instance, the yield loss prevented by an adapted cultivar could be even greater when combined with an appropriate irrigation treatment.

In fact, many of the identified trade-offs occur at nested temporal and spatial resolutions (e.g., short-term vs. long-term effects of cover crop, irrigation practices vs. regional water availability, planting choice vs. local, or national governance) that have hardly been captured by previous evaluation studies. With regard to time scale, the spatial expansion of long-term adaptation (e.g., cultivars, planting density) is limited by the vineyard renewal, which is estimated at around 2 to 3% in France (Agreste, 2018), whereas the adoption of short-term adaptations depends on the infra-annual organization of farm labor. Moreover, climatic changes could be described at a century scale (global warming) when dramatic events may occur at the scale of a few hours or days (heavy rain, heat waves). With regard to spatial scale, the close link between viticulture and *terroir* means that a wide range of spatial factors must be considered—soil, microclimate, and socio-economic (“Protected designation of Origin” areas, farm size, etc.)—when designing and evaluating adaptation strategies.

The design and implementation of effective combinations of adaptations require a quantification of the possible impacts of climate change, coupled with the sensitivity of those impacts to different adaptation activities (Diffenbaugh et al., 2011). Models may play a central role in managing various time steps and spatial units. Previous works dealing with adaptation have developed modeling tools with the aim of integrating climate projection into grapevine crop models (Moriondo et al., 2015). Models exist for some specific processes: WaLIS for water balance (Celette et al., 2010), VitiSim for carbon balance (Mirás-Avalos et al., 2018), NVINE for nitrogen cycle (Nendel and Kersebaum, 2004), STICS for yield (Fraga et al., 2018), among others. The few studies that integrated decision-making into their models are based on agent-based modeling (Delay et al., 2015; Tissot et al., 2017). Other decision models developed in viticulture could be adapted to climate change studies: VERDI (Ripoche et al., 2011), or DHIVINE (Martin-Clouaire et al., 2016). However, Corbeels et al. (2018) recently challenged the ability of crop models driven by climate model projections to identify promising adaptation, given the large uncertainties of model predictions.

In addition, the contribution of stakeholders is important in characterizing and considering local constraints and opportunities. The example of co-design and evaluation studies oriented toward the reduction of pesticide use offers promising tools (Lafond and Métral, 2015; Thiollet-Scholtus and Bockstaller, 2015). In fact, strategies of adaptation to climate change with the participation of stakeholders have already been evaluated (Battaglini et al., 2009; Nicholas and Durham, 2012; Alonso and Liu, 2013; Lereboullet et al., 2013). However, the quantitative evaluation or comparison of co-designed strategies under future climatic conditions has not yet been developed. Further researches need to be conducted in order to combine the co-design of spatial adaptation strategies with their quantitative evaluation under future climatic conditions.

### Insight for Developing a New Evaluation Framework

**Figure 9:**
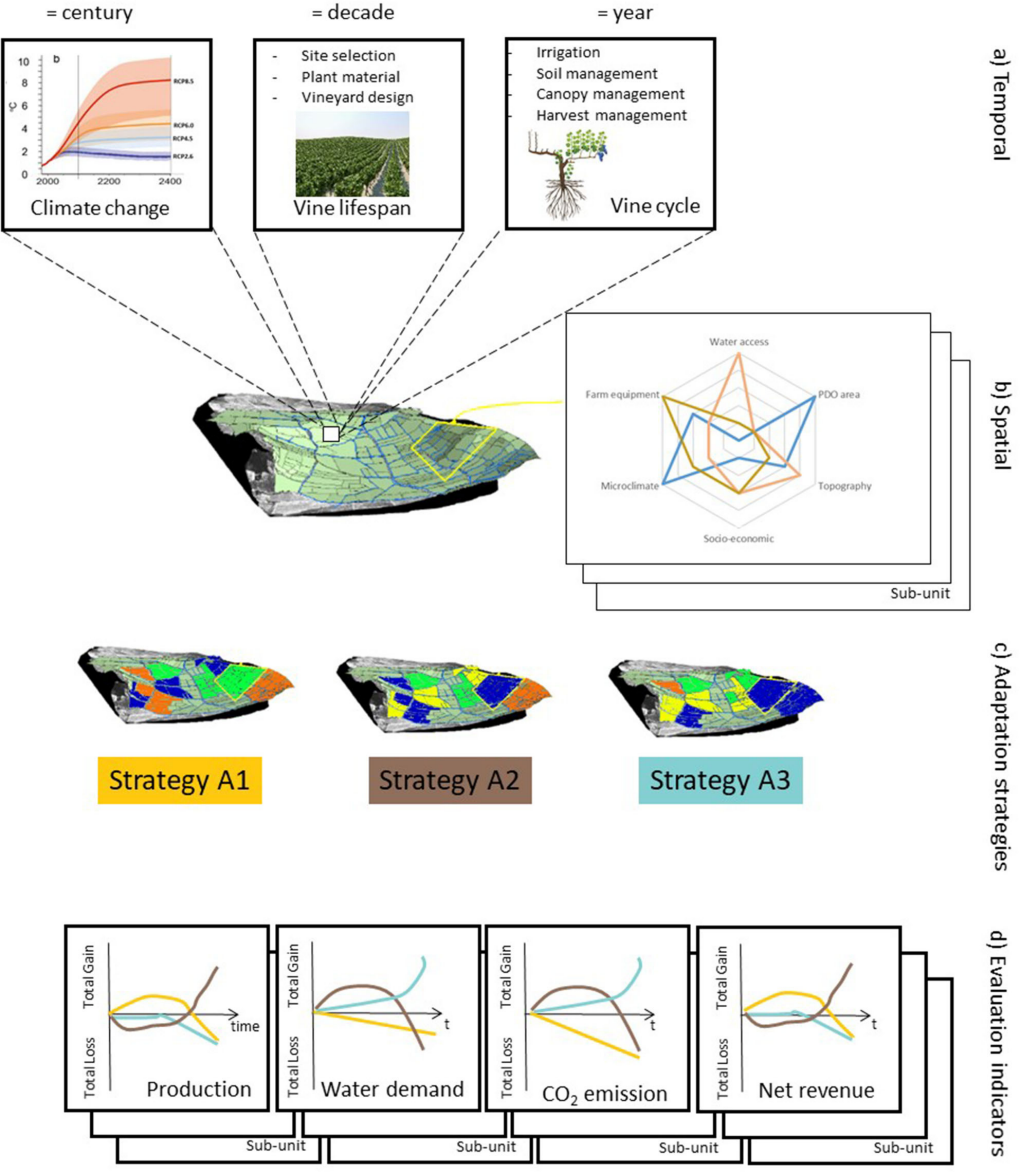
Conceptual diagram of the spatio-temporal model needed to design and evaluate strategies for adaptation to climate change across viticultural regions: **(A)** temporal scale integration and associated adaptation levers; **(B)** spatial factors; **(C)** exploration of adaptation strategies; and **(D)** calculation of evaluation indicators. In yellow, blue, and brown, three adaptation strategies that consist of three different spatial combinations of adaptation levers (in green, orange, and dark blue).

Based on the lessons learnt from reforestation studies (Cunningham et al., 2015), we propose a new framework of adaptation evaluation in four steps, considering different time and space scales, with a few to building spatially explicit strategies (Figure 9). A first step concerns the integration of three temporal scales (year, decade, century). A second step integrates spatial factors into the evaluation processes (water access, Protected Denomination of Origin areas, microclimate, etc.). A third step explores the spatialized adaptation strategies, considering a combination of adaptation in both time and space. A fourth step allows trade-offs to be identified by calculating multiple evaluation indicators over time.

The first step in evaluating local adaptation strategies may help researchers in considering the impact of climate change and of adaptation strategies over relevant times scales (year to century) (Figure 9A). Adequate models exist but they are far from being exhaustive (e.g., high temperature, CO_2_ effects) and parameterized for various contexts [soil, climate, cultivar, etc.)] (Moriondo et al., 2015). The existing models could be improved by conducting more focused research (experimental or on-farm), particularly in traditional grapevine systems (low density, traditional cultivars, and crop management). Other improvements lie in considering multi-year processes (e.g., mortality). Given the urgency of adaptation, expert opinion might also be used to develop and parameterize models when quantitative knowledge is unavailable. Close collaboration between researchers and winegrowers might help in designing better adaptation trials in order to fill knowledge gaps.

The second step consists in delimiting spatial sub-units that represent regions where the conditions of adaptation to climate change can be expected to be similar. The collection of data to parameterize models in each spatial sub-unit is laborious. The relevancy of required data (e.g., slope, soil type, water access, “Protected Designation of Origin” area) could be discussed with experts or local stakeholders. To go further, models should be scaled up to larger sub-units (farm, small agricultural region, catchment, country, etc.) (Figure 9B). This scaling up process requires spatial and temporal modeling methods that predict the aggregated effects of adaptation.

The third step, the integration of a detailed understanding of the plant and field processes with regional-scale modeling is a key toward predicting the effects of the spatial distribution of adaptation levers while considering biophysical and socio-economic diversity. The use of large-scale spatial and temporal models makes possible the exploration of a large range of plausible adaptation strategies, including future climate evolution (e.g., more frequent droughts, higher temperatures), economic choices (e.g., expansion of PDO areas, marketing labels, water prices) and social changes (e.g., consumer preferences) (Figure 9C).

In the final step, such models may be used to quantify a large range of evaluation indicators (environmental, economic, agronomic, etc.) in order to reveal trade-offs and avoid potential deleterious adaptation strategies (e.g., unbalanced water demand and supply, yield reduction, climate change mitigation) (Figure 9D). Evaluation indicators should be calculated across time as a beneficial strategy could appear as a mal-adaptation under future climatic conditions or, on the contrary, an apparently disadvantageous strategy could appear beneficial in the near future. The development of indicators should meet the objectives of various local stakeholders (wine-growers, policy-makers, environmental defenders, etc.).

In conclusion, rigorous evaluation of adaptation strategies for climate change helps to identify site-specific adaptation trade-offs. We argue that the development of methodologies to evaluate adaptation strategies, considering both complementary adaptations and scales, is essential to propose relevant information to decision-makers in the winegrowing sector. The development of spatial and temporal evaluation tools based on mixed knowledge—local and scientific—about grapevine response to climatic conditions, is a key for deciding how to locally adapt viticulture to climate change.

## Data Availability Statement

The raw data supporting the conclusions of this article will be made available by the authors, without undue reservation.

## Author Contributions

AN lead the systematic bibliographic research (problematic, equation search, metadata extraction). Articles selection procedure and criteria of analysis have been defined by AN, CG, LP, and LH. LH ensured the consistency of the method with her experience in dealing with PRISMA flows diagram. AN has processed to the reading and selection. The final article pool has been discussed and validated by the AN, CG, LP, and LH. AN organized and wrote the article with frequent interactions with the CG, LP, and LH. All authors contributed to the article and approved the submitted version.

## Conflict of Interest

The authors declare that the research was conducted in the absence of any commercial or financial relationships that could be construed as a potential conflict of interest.
