# Supplementary material for: Evaluating Strategies for Adaptation to Climate Change in Grapevine Production–A Systematic Review
Source: Front Plant Sci. 2021 Jan 14;11:607859. doi: 10.3389/fpls.2020.607859 (PMC7840846; doi:10.3389/fpls.2020.607859)
Supplement: Supplementary file 1 [file Table_1.DOCX]

Supplementary Material

# Supplementary Tables

**Supplementary Table 1.** Complete list of articles dataset (n=111) with description of the studied area, the adaptation (LT1: Site Selection, LT2: Plant Material, LT3: Vineyard design, LT4: Farm strategy, ST1: Irrigation, ST2: Soil management, ST3: Canopy management, ST4: Harvest and post-harvest management), scale and method.

| N° | Reference | Studied area | Adaptation | Scale | Method | N° | Reference | Studied area | Adaptation | Scale | Method |
| --- | --- | --- | --- | --- | --- | --- | --- | --- | --- | --- | --- |
| 1 | (Alonso and Liu, 2013) | Spain | LT1, LT4,ST1, ST4 | Farm | Expert judgement | **56** | (Lereboullet et al., 2013) | France, Australia | LT3, ST3, ST2, ST1, LT2, LT4 | Region | Expert judgement |
| 2 | (dos Santos et al., 2007) | Portugal | ST1 | Field | Experimental | **57** | (Levy and Lubell, 2018) | USA | LT1 | Region | Expert judgement |
| 3 | (Ramos and Martínez-Casasnovas, 2010) | Spain | LT3 | Field | Experimental | **58** | (Caravia et al., 2016) | Australia | LT3 | Field | Experimental |
| 4 | (Grantham et al., 2010) | USA | ST1 | Region | Modeling | **59** | (Caravia et al., 2017) | Australia | ST1 | Field | Experimental |
| 5 | (Bardsley et al., 2018) | Australia | LT2, LT4, ST1, ST2, ST3 | Region / Farm | Expert judgement | **60** | (Carvalho et al., 2017) | Portugal | LT2 | Plant | Experimental |
| 6 | (Kapur et al., 2007) | Italy | ST1 | Field / Region | Modeling | **61** | (Mirás-Avalos et al., 2016) | Spain | ST1 | Field | Experimental |
| 7 | (Battaglini et al., 2009) | Italy | ST1, LT2 | Field / Farm | Expert judgement | **62** | (Cirigliano et al., 2017) | Italy |  | Field | Experimental |
| 8 | (Belliveau et al., 2006) | Canada | ST1, LT4, ST3, LT2 | Farm | Expert judgement | **63** | (Medrano et al., 2015) | Spain |  | Plant | Review |
| 9 | (Diffenbaugh et al., 2011) | USA | LT1, LT2 | Region | Modeling | **64** | (Georgopoulou et al., 2017) | Greece |  | Farm/Region | Modeling |
| 10 | (Carvalho et al., 2018) | Portugal | ST1, LT2 | Field | Experimental | **65** | (Santesteban et al., 2017) | Spain | ST1, ST2 | Field | Experimental |
| 11 | (Bernardo et al., 2018) | Portugal | LT3, ST3, ST2, ST1, LT1, LT2 | Field | Review | **66** | (Martínez-Lüscher et al., 2017) | Spain | LT3 | Field | Experimental |
| 12 | (Duchene et al., 2012) | France | LT2 | Plant | Experimental | **67** | (Mirás-Avalos et al., 2018) | Spain | ST1 | Plant / Field | Modeling |
| 13 | (Nicholas and Durham, 2012) | USA | LT1, LT2, ST3, LT3, ST1 | Farm | Expert judgement | **68** | (Neethling et al., 2017) | France | ST3, ST2, ST1, LT2 | Field / Farm | Expert judgement |
| 14 | (Pieri et al., 2012) | France | LT2, LT3, LT1, ST1 | Field | Modeling | **69** | (Mosedale et al., 2016) | UK | LT1, LT2, LT3, LT4, ST1, ST2, ST3 | Plant/Region | Review |
| 15 | (Hannah et al., 2013) | USA | LT1 | Plant / Region | Modeling | **70** | (Paciello et al., 2017) | Italy | ST1 | Field | Experimental |
| 16 | (Milla‐Tapia et al., 2013) | Chile | LT2 | Plant | Experimental | **71** | (Pagliarani et al., 2017) | Italy | LT2 | Plant | Experimental |
| 17 | (Palliotti et al., 2013) | Italy | ST3 | Field | Experimental | **72** | (Petrie et al., 2017) | Australia | ST3 | Field | Experimental |
| 18 | (Raymond and Spoehr, 2013) | Australia | ST2, ST1, LT2 | Farm | Expert judgement | **73** | (Ronco et al., 2017) | Italy | ST1 | Region | Expert judgement |
| 19 | (Stoll et al., 2013) | Germany | ST3 | Field | Experimental | **74** | (Sacchelli et al., 2016) | Italy | ST1, LT3, LT2, LT4 | Farm | Expert judgement |
| 20 | (Attia et al., 2014) | France | ST3 | Plant | Experimental | **75** | (Tissot et al., 2017) | France | - | Plant/Field/Region | Expert judgement |
| 21 | (Clingeleffer, 2010) | Australia | LT3, ST1, LT2, ST3 | Field | Review | **76** | (Palliotti et al., 2014) | Italy | ST3, LT3 | Plant/Region | Review |
| 22 | (Baronti et al., 2014) | Italy | ST2 | Field | Experimental | **77** | (Tomaz et al., 2017) | Portugal | ST1, ST2 | Field | Experimental |
| 23 | (Fuhrer et al., 2014) | Suisse | ST1 | Region | Modeling | **78** | (Torres et al., 2017) | Spain | ST1, LT2 | Plant | Experimental |
| 24 | (Londo and Johnson, 2014) | USA | LT2 | Plant | Experimental | **79** | (Bigard et al., 2018) | France | LT2 | Plant | Experimental |
| 25 | (Costa et al., 2016) | Portugal | ST1, ST2 | Plant/Field/Region | Review | **80** | (Bonada et al., 2018) | Australia | ST1 | Field | Experimental |
| 26 | (Meggio et al., 2014) | Italy | LT2 | Plant | Experimental | **81** | (Poni et al., 2018) | Italy | ST3 | Field | Review |
| 27 | (Pickering et al., 2014) | Canada | LT4 | Sector | Expert judgement | **82** | (Chrysargyris et al., 2018) | Cyprus | ST1, ST2 | Field | Experimental |
| 28 | (Șerdinescu et al., 2014) | Romania | ST2, ST3 | Field | Experimental | **83** | (De Micco et al., 2018) | Italy | LT2 | Plant | Experimental |
| 29 | (Dequin et al., 2017) | France | ST4 | Farm | Review | **84** | (Dinis et al., 2018b). | Portugal | ST3 | Plant | Experimental |
| 30 | (de C. Teixeira et al., 2014) | Brazil | ST3 | Plant | Modeling | **85** | (Dinis et al., 2018a) | Portugal | ST3 | Plant | Experimental |
| 31 | (Berdeja et al., 2015) | France | LT2 | Plant | Experimental | **86** | (Fraga et al., 2018a) | Portugal | ST1 | Field/Region | Modeling |
| 32 | (Corso et al., 2015) | Italy | LT2 | Plant | Modeling | **87** | (Fraga and Santos, 2018) | Portugal | ST2 | Field/Region | Modeling |
| 33 | (Delay et al., 2015) | France | LT1, LT4 | Region | Expert judgement | **88** | (Franques et al., 2018) | Spain | ST4 | Farm | Experimental |
| 34 | (Duchene, 2016) | France | LT2 | Plant | Review | **89** | (Gil et al., 2018) | Chile | ST1, LT3, ST2 | Field | Experimental |
| 35 | (Genesio et al., 2015) | Italy | ST2 | Field | Experimental | **90** | (Malacarne et al., 2018) | Italy | LT2 | Plant | Experimental |
| 36 | (Lobos et al., 2015) | Chile | ST3 | Field | Experimental | **91** | (Phogat et al., 2018) | Australia | ST1 | Region | Modeling |
| 37 | (Fleming et al., 2015) | Australia | - | Farm | Expert judgement | **92** | (Reshef et al., 2018) | Israel | LT3 | Plant | Experimental |
| 38 | (Flexas et al., 2010) | Spain | LT2, ST1 | Plant | Review | **93** | (Serra et al., 2014) | South Africa | LT2 | Plant | Review |
| 39 | (Trigo-Córdoba et al., 2015) | Spain | ST1 | Plant | Experimental | **94** | (Simonneau et al., 2017) | France | LT2 | Plant | Review |
| 40 | (Carvalho-Santos et al., 2016) | Portugal | LT1 | Region | Modeling | **95** | (Romero et al., 2018) | Spain | ST1, LT2 | Plant | Experimental |
| 41 | (Ramos, 2016) | Spain | LT3 | Region | Modeling | **96** | (Savi et al., 2018) | Italy | ST1 | Field | Experimental |
| 42 | (Dal Santo et al., 2016) | Italy | LT2 | Plant | Experimental | **97** | (Schelezki et al., 2018) | Australia | ST4 | Farm | Experimental |
| 43 | (Galbreath, 2011) | Australia | ST1 | Farm | Expert judgement | **98** | (Tomás et al., 2014) | Spain | ST3, LT2 | Plant | Review |
| 44 | (Galbreath, 2014) | Australia | ST1 | Farm | Expert judgement | **99** | (Torres et al., 2018a) | Spain | ST1, ST2 | Plant | Experimental |
| 45 | (Galbreath et al., 2016) | Australia | LT2, ST3, LT3, LT1, ST1 | Farm | Expert judgement | **100** | (Torres et al., 2018b) | Spain | ST2 | Plant | Experimental |
| 46 | (Hopper et al., 2016) | USA | LT2 | Plant | Experimental | **101** | (Wenter et al., 2018) | Italy | ST1 | Field | Experimental |
| 47 | (Hunter et al., 2016) | South Africa | LT3 | Plant / Field | Experimental | **102** | (Aparicio et al., 2019) | Malta | ST1 | Field / Farm | Modeling |
| 48 | (Montana et al., 2016) | Argentina | LT1, ST1 | Region | Experimental | **103** | (Buesa et al., 2019) | Spain | ST3 | Field | Experimental |
| 49 | (Olen et al., 2016) | USA | LT1, ST1 | Farm | Modeling | **104** | (Trouvelot et al., 2015) | France | ST2 | Field | Review |
| 50 | (Resco et al., 2016) | Spain | ST1, LT2,LT3 | Region | Modeling | **105** | (Fahey and Rogiers, 2019) | Australia | ST3 | Plant | Experimental |
| 51 | (Holland and Smit, 2014) | Canada | ST3, LT1, LT2 | Farm | Expert judgement | **106** | (van Leeuwen and Darriet, 2016) | France | LT1, LT2, LT3, ST3 | Sector | Review |
| 52 | (Vaz et al., 2016) | Portugal | LT2 | Field | Experimental | **107** | (Molitor et al., 2019) | Luxembourg | LT3 | Field | Experimental |
| 53 | (Zhu et al., 2016) | Italy | LT1, LT2 | Field/Farm/Region | Modeling | **108** | (Sabir, 2016) | Turkey | ST2 | Plant | Experimental |
| 54 | (Amendola et al., 2017) | Italy | ST2 | Field | Experimental | **109** | (Wolkovich et al., 2017) | USA | LT1, LT2 | Plant/Region | Review |
| 55 | (Keller, 2010) | USA | ST1, LT3, ST2, ST3, LT1 | Field/Region | Review | **110** | (Zhang et al., 2016) | France | LT2 | Plant | Review |
|  |  |  |  |  |  | **111** | (Valentini et al., 2019) | Italy | ST3 | Field | Experimental |

**Bibliography :**

Alonso, A. D., and Liu, Y. (2013). Climate Change in the Wine Sector of an Ultra‐Peripheral European Region: A Case Study. *Agroecology and Sustainable Food Systems* 37, 291–315. doi:10.1080/10440046.2012.712089.

Amendola, C., Montagnoli, A., Terzaghi, M., Trupiano, D., Oliva, F., Baronti, S., et al. (2017). Short-term effects of biochar on grapevine fine root dynamics and arbuscular mycorrhizae production. *Agriculture, Ecosystems & Environment* 239, 236–245. doi:10.1016/j.agee.2017.01.025.

Aparicio, J., Tenza-Abril, A. J., Borg, M., Galea, J., and Candela, L. (2019). Agricultural irrigation of vine crops from desalinated and brackish groundwater under an economic perspective. A case study in Siġġiewi, Malta. *Science of The Total Environment* 650, 734–740. doi:10.1016/j.scitotenv.2018.09.059.

Attia, F., Martinez, L., and Lamaze, T. (2014). Foliar application of processed calcite particles improves leaf photosynthesis of potted <em>Vitis vinifera</em> L. (var. ‘Cot’) grown under water deficit. *1* 48, 237–245. doi:10.20870/oeno-one.2014.48.4.1691.

Bardsley, D. K., Palazzo, E., and Pütz, M. (2018). Regional path dependence and climate change adaptation: A case study from the McLaren Vale, South Australia. *Journal of Rural Studies* 63, 24–33. doi:10.1016/j.jrurstud.2018.08.015.

Baronti, S., Vaccari, F. P., Miglietta, F., Calzolari, C., Lugato, E., Orlandini, S., et al. (2014). Impact of biochar application on plant water relations in Vitis vinifera (L.). *European Journal of Agronomy* 53, 38–44. doi:10.1016/j.eja.2013.11.003.

Battaglini, A., Barbeau, G., Bindi, M., and Badeck, F.-W. (2009). European winegrowers’ perceptions of climate change impact and options for adaptation. *Regional Environmental Change* 9, 61–73. doi:10.1007/s10113-008-0053-9.

Belliveau, S., Smit, B., and Bradshaw, B. (2006). Multiple exposures and dynamic vulnerability: Evidence from the grape industry in the Okanagan Valley, Canada. *Global Environmental Change* 16, 364–378. doi:10.1016/j.gloenvcha.2006.03.003.

Berdeja, M., Nicolas, P., Kappel, C., Dai, Z. W., Hilbert, G., Peccoux, A., et al. (2015). Water limitation and rootstock genotype interact to alter grape berry metabolism through transcriptome reprogramming. *Hortic Res* 2, 15012. doi:10.1038/hortres.2015.12.

Bernardo, S., Dinis, L.-T., Machado, N., and Moutinho-Pereira, J. (2018). Grapevine abiotic stress assessment and search for sustainable adaptation strategies in Mediterranean-like climates. A review. *Agronomy for Sustainable Development* 38. doi:10.1007/s13593-018-0544-0.

Bigard, A., Berhe, D. T., Maoddi, E., Sire, Y., Boursiquot, J.-M., Ojeda, H., et al. (2018). Vitis vinifera L. Fruit Diversity to Breed Varieties Anticipating Climate Changes. *Front. Plant Sci.* 9, 455. doi:10.3389/fpls.2018.00455.

Bonada, M., Buesa, I., Moran, M. A., and Sadras, V. O. (2018). Interactive effects of warming and water deficit on Shiraz vine transpiration in the Barossa Valley, Australia. *OENE One* 52. doi:10.20870/oeno-one.2018.52.2.2141.

Buesa, I., Caccavello, G., Basile, B., Merli, M. C., Poni, S., Chirivella, C., et al. (2019). Delaying berry ripening of Bobal and Tempranillo grapevines by late leaf removal in a semi-arid and temperate-warm climate under different water regimes: Late leaf removal effects in Bobal and Tempranillo. *Australian Journal of Grape and Wine Research* 25, 70–82. doi:10.1111/ajgw.12368.

Caravia, L., Collins, C., Petrie, P. R., and Tyerman, S. D. (2016). Application of shade treatments during Shiraz berry ripening to reduce the impact of high temperature: Shade reduces impact of high temperature on Shiraz. *Australian Journal of Grape and Wine Research* 22, 422–437. doi:10.1111/ajgw.12248.

Caravia, L., Pagay, V., Collins, C., and Tyerman, S. D. (2017). Application of sprinkler cooling within the bunch zone during ripening of Cabernet Sauvignon berries to reduce the impact of high temperature: Sprinkler cooling of Cabernet Sauvignon. *Australian Journal of Grape and Wine Research* 23, 48–57. doi:10.1111/ajgw.12255.

Carvalho, L. C., Coito, J. L., Gonçalves, E. F., Lopes, C., and Amâncio, S. (2018). Physiological and agronomical responses to environmental fluctuations of two Portuguese grapevine varieties during three field seasons. *Ciência Téc. Vitiv.* 33, 1–14. doi:10.1051/ctv/20183301001.

Carvalho, L. C., Silva, M., Coito, J. L., Rocheta, M. P., and Amâncio, S. (2017). Design of a Custom RT-qPCR Array for Assignment of Abiotic Stress Tolerance in Traditional Portuguese Grapevine Varieties. *Front. Plant Sci.* 8. doi:10.3389/fpls.2017.01835.

Carvalho-Santos, C., Nunes, J. P., Monteiro, A. T., Hein, L., and Honrado, J. P. (2016). Assessing the effects of land cover and future climate conditions on the provision of hydrological services in a medium-sized watershed of Portugal: Impacts of Land Cover and Future Climate on Hydrological Services. *Hydrological Processes* 30, 720–738. doi:10.1002/hyp.10621.

Chrysargyris, A., Xylia, P., Litskas, V., Mandoulaki, A., Antoniou, D., Boyias, T., et al. (2018). Drought stress and soil management practices in grapevines in Cyprus under the threat of climate change. *Journal of Water and Climate Change*. doi:10.2166/wcc.2018.135.

Cirigliano, P., Vincenza Chiriacò, M., CMCC – Fondazione Centro Euro-Mediterraneo sui Cambiamenti Climatici, Nunez, A., Universidad Autonoma de Chihuahua, Dal Monte, G., et al. (2017). Efecto combinado de la aplicación de riego y compost sobre la composición de la baya Montepulciano en un entorno volcánico de la región de Lacio (Italia central). *Ciencia e investigación agraria* 44, 195–206. doi:10.7764/rcia.v44i2.1691.

Clingeleffer, P. R. (2010). Plant management research: status and what it can offer to address challenges and limitations. *Australian Journal of Grape and Wine Research* 16, 25–32. doi:10.1111/j.1755-0238.2009.00075.x.

Concepción Ramos, M. (2016). Soil losses in rainfed Mediterranean vineyards under climate change scenarios. The effects of drainage terraces. *AIMS Agriculture and Food* 1, 124–143. doi:10.3934/agrfood.2016.2.124.

Corso, M., Vannozzi, A., Maza, E., Vitulo, N., Meggio, F., Pitacco, A., et al. (2015). Comprehensive transcript profiling of two grapevine rootstock genotypes contrasting in drought susceptibility links the phenylpropanoid pathway to enhanced tolerance. *EXBOTJ* 66, 5739–5752. doi:10.1093/jxb/erv274.

Costa, J. M., Vaz, M., Escalona, J., Egipto, R., Lopes, C., Medrano, H., et al. (2016). Modern viticulture in southern Europe: Vulnerabilities and strategies for adaptation to water scarcity. *Agricultural Water Management* 164, 5–18. doi:10.1016/j.agwat.2015.08.021.

Dal Santo, S., Palliotti, A., Zenoni, S., Tornielli, G. B., Fasoli, M., Paci, P., et al. (2016). Distinct transcriptome responses to water limitation in isohydric and anisohydric grapevine cultivars. *BMC Genomics* 17, 815. doi:10.1186/s12864-016-3136-x.

de C. Teixeira, A. H., Tonietto, J., Pereira, G. E., Hernandez, F. B. T., Angelotti, F., and Lopes, H. L. (2014). AGRO-CLIMATIC SUITABILITY DELIMITATION FOR TABLE AND WINE GRAPE CROPS UNDER IRRIGATION CONDITIONS IN NORTHEASTERN BRAZIL. *Acta Hortic.*, 277–286. doi:10.17660/ActaHortic.2014.1038.33.

De Micco, V., Zalloni, E., Battipaglia, G., Erbaggio, A., Scognamiglio, P., Caputo, R., et al. (2018). Rootstock effect on tree-ring traits in grapevine under a climate change scenario. *IAWA Journal* 39, 145–155. doi:10.1163/22941932-20170199.

Delay, E., Piou, C., and Quenol, H. (2015). The mountain environment, a driver for adaptation to climate change. *Land Use Policy* 48, 51–62. doi:10.1016/j.landusepol.2015.05.008.

Dequin, S., Escudier, J.-L., Bely, M., Noble, J., Albertin, W., Masneuf-Pomarède, I., et al. (2017). How to adapt winemaking practices to modified grape composition under climate change conditions. *1* 51, 205–214. doi:10.20870/oeno-one.2017.51.2.1584.

Diffenbaugh, N. S., White, M. A., Jones, G. V., and Ashfaq, M. (2011). Climate adaptation wedges: a case study of premium wine in the western United States. *Environ. Res. Lett.* 6, 024024. doi:10.1088/1748-9326/6/2/024024.

Dinis, L.-T., Bernardo, S., Luzio, A., Pinto, G., Meijón, M., Pintó-Marijuan, M., et al. (2018a). Kaolin modulates ABA and IAA dynamics and physiology of grapevine under Mediterranean summer stress. *Journal of Plant Physiology* 220, 181–192. doi:10.1016/j.jplph.2017.11.007.

Dinis, L.-T., Malheiro, A. C., Luzio, A., Fraga, H., Ferreira, H., Gonçalves, I., et al. (2018b). Improvement of grapevine physiology and yield under summer stress by kaolin-foliar application: water relations, photosynthesis and oxidative damage. *Photosynthetica* 56, 641–651. doi:10.1007/s11099-017-0714-3.

dos Santos, T. P., Lopes, C. M., Lucília Rodrigues, M., de Souza, C. R., Ricardo-da-Silva, J. M., Maroco, J. P., et al. (2007). Effects of deficit irrigation strategies on cluster microclimate for improving fruit composition of Moscatel field-grown grapevines. *Scientia Horticulturae* 112, 321–330. doi:10.1016/j.scienta.2007.01.006.

Duchene, E. (2016). How can grapevine genetics contribute to the adaptation to climate change? *OENO One* 50. doi:10.20870/oeno-one.2016.50.3.98.

Duchene, E., Butterlin, G., Dumas, V., and Merdinoglu, D. (2012). Towards the adaptation of grapevine varieties to climate change: QTLs and candidate genes for developmental stages. *Theor. Appl. Genet.* 124, 623–635. doi:10.1007/s00122-011-1734-1.

Fahey, D. J., and Rogiers, S. Y. (2019). Di-1-p-menthene reduces grape leaf and bunch transpiration. *Australian Journal of Grape and Wine Research* 25, 134–141. doi:10.1111/ajgw.12371.

Fleming, A., Rickards, L., and Dowd, A.-M. (2015). Understanding convergence and divergence in the framing of climate change responses: An analysis of two wine companies. *Environmental Science & Policy* 51, 202–214. doi:10.1016/j.envsci.2015.04.003.

Flexas, J., GalmãS, J., Gallã, A., GulãAs, J., Pou, A., Ribas-Carbo, M., et al. (2010). Improving water use efficiency in grapevines: potential physiological targets for biotechnological improvement. *Australian Journal of Grape and Wine Research* 16, 106–121. doi:10.1111/j.1755-0238.2009.00057.x.

Fraga, H., García de Cortázar Atauri, I., and Santos, J. A. (2018a). Viticultural irrigation demands under climate change scenarios in Portugal. *Agricultural Water Management* 196, 66–74. doi:10.1016/j.agwat.2017.10.023.

Fraga, H., Pinto, J. G., and Santos, J. A. (2018b). Climate change projections for chilling and heat forcing conditions in European vineyards and olive orchards: a multi-model assessment. *Climatic Change*. doi:10.1007/s10584-018-2337-5.

Fraga, H., and Santos, J. A. (2018). Vineyard mulching as a climate change adaptation measure: Future simulations for Alentejo, Portugal. *Agricultural Systems* 164, 107–115. doi:10.1016/j.agsy.2018.04.006.

Franques, J., Araque, I., El Khoury, M., Lucas, P. M., Reguant, C., and Bordons, A. (2018). Selection and characterization of autochthonous strains of Oenococcus oeni for vinification in Priorat (Catalonia, Spain). *OENE One* 52, 45–56. doi:10.20870/oeno-one.2018.52.1.1908.

Fuhrer, J., Smith, P., and Gobiet, A. (2014). Implications of climate change scenarios for agriculture in alpine regions — A case study in the Swiss Rhone catchment. *Science of The Total Environment* 493, 1232–1241. doi:10.1016/j.scitotenv.2013.06.038.

Galbreath, J. (2011). To What Extent is Business Responding to Climate Change? Evidence from a Global Wine Producer. *J Bus Ethics* 104, 421–432. doi:10.1007/s10551-011-0919-5.

Galbreath, J. (2014). Climate Change Response: Evidence from the Margaret River Wine Region of Australia. *Business Strategy and the Environment* 23, 89–104. doi:10.1002/bse.1762.

Galbreath, J., Charles, D., and Oczkowski, E. (2016). The Drivers of Climate Change Innovations: Evidence from the Australian Wine Industry. *J Bus Ethics* 135, 217–231. doi:10.1007/s10551-014-2461-8.

Genesio, L., Miglietta, F., Baronti, S., and Vaccari, F. P. (2015). Biochar increases vineyard productivity without affecting grape quality: Results from a four years field experiment in Tuscany. *Agriculture, Ecosystems & Environment* 201, 20–25. doi:10.1016/j.agee.2014.11.021.

Georgopoulou, E., Mirasgedis, S., Sarafidis, Y., Vitaliotou, M., Lalas, D. P., Theloudis, I., et al. (2017). Climate change impacts and adaptation options for the Greek agriculture in 2021–2050: A monetary assessment. *Climate Risk Management* 16, 164–182. doi:10.1016/j.crm.2017.02.002.

Gil, P. M., Lobos, P., Durán, K., Olguín, J., Cea, D., and Schaffer, B. (2018). Partial root-zone drying irrigation, shading, or mulching effects on water savings, productivity and quality of ‘Syrah’ grapevines. *Scientia Horticulturae* 240, 478–483. doi:10.1016/j.scienta.2018.06.050.

Grantham, T. E., Merenlender, A. M., and Resh, V. H. (2010). Climatic influences and anthropogenic stressors: an integrated framework for streamflow management in Mediterranean-climate California, USA. *Freshw. Biol.* 55, 188–204. doi:10.1111/j.1365-2427.2009.02379.x.

Hannah, L., Roehrdanz, P. R., Ikegami, M., Shepard, A. V., Shaw, M. R., Tabor, G., et al. (2013). Climate change, wine, and conservation. *Proceedings of the National Academy of Sciences* 110, 6907–6912.

Holland, T., and Smit, B. (2014). Recent climate change in the Prince Edward County winegrowing region, Ontario, Canada: implications for adaptation in a fledgling wine industry. *Reg Environ Change* 14, 1109–1121. doi:10.1007/s10113-013-0555-y.

Hopper, D. W., Ghan, R., Schlauch, K. A., and Cramer, G. R. (2016). Transcriptomic network analyses of leaf dehydration responses identify highly connected ABA and ethylene signaling hubs in three grapevine species differing in drought tolerance. *BMC Plant Biology* 16, 118. doi:10.1186/s12870-016-0804-6.

Hunter, J. J., Volschenk, C. G., and Zorer, R. (2016). Vineyard row orientation of Vitis vinifera L. cv. Shiraz/101-14 Mgt: Climatic profiles and vine physiological status. *Agricultural and Forest Meteorology* 228–229, 104–119. doi:10.1016/j.agrformet.2016.06.013.

Kapur, B., Steduto, P., and Todorovic, M. (2007). Prediction of Climatic Change for the Next 100 Years in the Apulia Region, Southern Italy. *Ital J Agronomy* 2, 365. doi:10.4081/ija.2007.365.

Keller, M. (2010). Managing grapevines to optimise fruit development in a challenging environment: a climate change primer for viticulturists. *Australian Journal of Grape and Wine Research* 16, 56–69. doi:10.1111/j.1755-0238.2009.00077.x.

Lereboullet, A.-L., Beltrando, G., and Bardsley, D. K. (2013). Socio-ecological adaptation to climate change: A comparative case study from the Mediterranean wine industry in France and Australia. *Agriculture, Ecosystems & Environment* 164, 273–285. doi:10.1016/j.agee.2012.10.008.

Levy, M. A., and Lubell, M. N. (2018). Innovation, cooperation, and the structure of three regional sustainable agriculture networks in California. *Reg Environ Change* 18, 1235–1246. doi:10.1007/s10113-017-1258-6.

Lobos, G. A., Acevedo-Opazo, C., Guajardo-Moreno, A., Valdés-Gómez, H., Taylor, J. A., and Laurie, V. F. (2015). Effects of kaolin-based particle film and fruit zone netting on Cabernet Sauvignon grapevine physiology and fruit quality. *1* 49, 137–144. doi:10.20870/oeno-one.2015.49.2.86.

Londo, J. P., and Johnson, L. M. (2014). Variation in the chilling requirement and budburst rate of wild Vitis species. *Environmental and Experimental Botany* 106, 138–147. doi:10.1016/j.envexpbot.2013.12.012.

Malacarne, G., Pilati, S., Valentini, S., Asnicar, F., Moretto, M., Sonego, P., et al. (2018). Discovering Causal Relationships in Grapevine Expression Data to Expand Gene Networks. A Case Study: Four Networks Related to Climate Change. *Front. Plant Sci.* 9. doi:10.3389/fpls.2018.01385.

Martínez-Lüscher, J., Chen, C. C. L., Brillante, L., and Kurtural, S. K. (2017). Partial Solar Radiation Exclusion with Color Shade Nets Reduces the Degradation of Organic Acids and Flavonoids of Grape Berry ( *Vitis vinifera* L.). *Journal of Agricultural and Food Chemistry* 65, 10693–10702. doi:10.1021/acs.jafc.7b04163.

Medrano, H., Tomás, M., Martorell, S., Escalona, J.-M., Pou, A., Fuentes, S., et al. (2015). Improving water use efficiency of vineyards in semi-arid regions. A review. *Agronomy for Sustainable Development* 35, 499–517. doi:10.1007/s13593-014-0280-z.

Meggio, F., Prinsi, B., Negri, A. S., Lorenzo, G. S. D., Lucchini, G., Pitacco, A., et al. (2014). Biochemical and physiological responses of two grapevine rootstock genotypes to drought and salt treatments. *Australian Journal of Grape and Wine Research* 20, 310–323. doi:10.1111/ajgw.12071.

Milla‐Tapia, A., Gómez, S., Moncada, X., León, P., Ibacache, A., Rosas, M., et al. (2013). Naturalised grapevines collected from arid regions in Northern Chile exhibit a high level of genetic diversity. *Australian Journal of Grape and Wine Research* 19, 299–310. doi:10.1111/ajgw.12020.

Mirás-Avalos, J. M., Trigo-Córdoba, E., Bouzas-Cid, Y., and Orriols-Fernández, I. (2016). Irrigation effects on the performance of grapevine (Vitis vinifera L.) cv. ‘Albariño’ under the humid climate of Galicia. *OENO One* 50. doi:10.20870/oeno-one.2016.50.4.63.

Mirás-Avalos, J. M., Uriarte, D., Lakso, A. N., and Intrigliolo, D. S. (2018). Modeling grapevine performance with ‘VitiSim’, a weather-based carbon balance model: Water status and climate change scenarios. *Scientia Horticulturae* 240, 561–571. doi:10.1016/j.scienta.2018.06.065.

Molitor, D., Schultz, M., Mannes, R., Pallez-Barthel, M., Hoffmann, L., and Beyer, M. (2019). Semi-Minimal Pruned Hedge: A Potential Climate Change Adaptation Strategy in Viticulture. *Agronomy* 9, 173. doi:10.3390/agronomy9040173.

Montana, E., Diaz, H. P., and Hurlbert, M. (2016). Development, local livelihoods, and vulnerabilities to global environmental change in the South American Dry Andes. *Reg. Envir. Chang.* 16, 2215–2228. doi:10.1007/s10113-015-0888-9.

Montaña, E., Diaz, H. P., and Hurlbert, M. (2016). Development, local livelihoods, and vulnerabilities to global environmental change in the South American Dry Andes. *Reg Environ Change* 16, 2215–2228. doi:10.1007/s10113-015-0888-9.

Mosedale, J. R., Abernethy, K. E., Smart, R. E., Wilson, R. J., and Maclean, I. M. D. (2016). Climate change impacts and adaptive strategies: lessons from the grapevine. *Global Change Biology* 22, 3814–3828. doi:10.1111/gcb.13406.

Neethling, E., Petitjean, T., Quénol, H., and Barbeau, G. (2017). Assessing local climate vulnerability and winegrowers’ adaptive processes in the context of climate change. *Mitigation and Adaptation Strategies for Global Change* 22, 777–803. doi:10.1007/s11027-015-9698-0.

Nicholas, K. A., and Durham, W. H. (2012). Farm-scale adaptation and vulnerability to environmental stresses: Insights from winegrowing in Northern California. *Global Environmental Change* 22, 483–494. doi:10.1016/j.gloenvcha.2012.01.001.

Olen, B., Wu, J., and Langpap, C. (2016). Irrigation Decisions for Major West Coast Crops: Water Scarcity and Climatic Determinants. *American Journal of Agricultural Economics* 98, 254–275. doi:10.1093/ajae/aav036.

Paciello, P., Mencarelli, F., Palliotti, A., Ceccantoni, B., Thibon, C., Darriet, P., et al. (2017). Nebulized water cooling of the canopy affects leaf temperature, berry composition and wine quality of Sauvignon blanc: Nebulized water cooling of the canopy affects wine quality of Sauvignon blanc. *J. Sci. Food Agric.* 97, 1267–1275. doi:10.1002/jsfa.7860.

Pagliarani, C., Vitali, M., Ferrero, M., Vitulo, N., Incarbone, M., Lovisolo, C., et al. (2017). The Accumulation of miRNAs Differentially Modulated by Drought Stress Is Affected by Grafting in Grapevine. *Plant Physiol* 173, 2180–2195. doi:10.1104/pp.16.01119.

Palliotti, A., Panara, F., Famiani, F., Sabbatini, P., Howell, G. S., Silvestroni, O., et al. (2013). Postveraison Application of Antitranspirant Di-1- *p* -Menthene to Control Sugar Accumulation in Sangiovese Grapevines. *Am. J. Enol. Vitic.* 64, 378–385. doi:10.5344/ajev.2013.13015.

Palliotti, A., Tombesi, S., Silvestroni, O., Lanari, V., Gatti, M., and Poni, S. (2014). Changes in vineyard establishment and canopy management urged by earlier climate-related grape ripening: A review. *Scientia Horticulturae* 178, 43–54. doi:10.1016/j.scienta.2014.07.039.

Petrie, P. R., Brooke, S. J., Moran, M. A., and Sadras, V. O. (2017). Pruning after budburst to delay and spread grape maturity. *Australian Journal of Grape and Wine Research* 23, 378–389. doi:10.1111/ajgw.12303.

Phogat, V., Cox, J. W., and Šimůnek, J. (2018). Identifying the future water and salinity risks to irrigated viticulture in the Murray-Darling Basin, South Australia. *Agricultural Water Management* 201, 107–117. doi:10.1016/j.agwat.2018.01.025.

Pickering, K., Plummer, R., and Pickering, G. (2014). DETERMINING ADAPTIVE CAPACITY TO CLIMATE CHANGE IN THE GRAPE AND WINE INDUSTRY. 7.

Pieri, P., Lebon, E., and Brisson, N. (2012). CLIMATE CHANGE IMPACT ON FRENCH VINEYARDS AS PREDICTED BY MODELS. *Acta Horticulturae*, 29–37. doi:10.17660/ActaHortic.2012.931.2.

Poni, S., Gatti, M., Palliotti, A., Dai, Z., Duchêne, E., Truong, T.-T., et al. (2018). Grapevine quality: A multiple choice issue. *Scientia Horticulturae* 234, 445–462. doi:10.1016/j.scienta.2017.12.035.

Ramos, M. C., and Martínez-Casasnovas, J. A. (2010). Soil water balance in rainfed vineyards of the Penedès region (Northeastern Spain) affected by rainfall characteristics and land levelling: influence on grape yield. *Plant Soil* 333, 375–389. doi:10.1007/s11104-010-0353-y.

Raymond, C. M., and Spoehr, J. (2013). The acceptability of climate change in agricultural communities: Comparing responses across variability and change. *Journal of Environmental Management* 115, 69–77. doi:10.1016/j.jenvman.2012.11.003.

Resco, P., Iglesias, A., Bardají, I., and Sotés, V. (2016). Exploring adaptation choices for grapevine regions in Spain. *Reg Environ Change* 16, 979–993. doi:10.1007/s10113-015-0811-4.

Reshef, N., Agam, N., and Fait, A. (2018). Grape Berry Acclimation to Excessive Solar Irradiance Leads to Repartitioning between Major Flavonoid Groups. *J. Agric. Food Chem.* 66, 3624–3636. doi:10.1021/acs.jafc.7b04881.

Romero, P., Botía, P., and Navarro, J. M. (2018). Selecting rootstocks to improve vine performance and vineyard sustainability in deficit irrigated Monastrell grapevines under semiarid conditions. *Agricultural Water Management* 209, 73–93. doi:10.1016/j.agwat.2018.07.012.

Ronco, P., Zennaro, F., Torresan, S., Critto, A., Santini, M., Trabucco, A., et al. (2017). A risk assessment framework for irrigated agriculture under climate change. *Advances in Water Resources* 110, 562–578. doi:10.1016/j.advwatres.2017.08.003.

Sabir, A. (2016). Vegetative and Reproductive Growth Responses of Grapevine cv. “Italia” (Vitis vinifera L.) Grafted on Different Rootstocks to Contrasting Soil Water Status. 13.

Sacchelli, S., Fabbrizzi, S., and Menghini, S. (2016). Climate Change, Wine and Sustainability: A Quantitative Discourse Analysis of the International Scientific Literature. *Agriculture and Agricultural Science Procedia* 8, 167–175. doi:10.1016/j.aaspro.2016.02.090.

Santesteban, L. G., Miranda, C., Urrestarazu, J., Loidi, M., and Royo, J. B. (2017). Severe trimming and enhanced competition of laterals as a tool to delay ripening in Tempranillo vineyards under semiarid conditions. *OENO One* 51, 191–203. doi:10.20870/oeno-one.2017.51.2.1583.

Savi, T., Petruzzellis, F., Martellos, S., Stenni, B., Dal Borgo, A., Zini, L., et al. (2018). Vineyard water relations in a karstic area: deep roots and irrigation management. *Agriculture, Ecosystems & Environment* 263, 53–59. doi:10.1016/j.agee.2018.05.009.

Schelezki, O. J., Smith, P. A., Hranilovic, A., Bindon, K. A., and Jeffery, D. W. (2018). Comparison of consecutive harvests versus blending treatments to produce lower alcohol wines from Cabernet Sauvignon grapes: Impact on polysaccharide and tannin content and composition. *Food Chemistry* 244, 50–59. doi:10.1016/j.foodchem.2017.10.024.

Șerdinescu, A., Pîrcălabu, L., and Fotescu, L. (2014). Influence of soil maintenance systems and fruit load on grapes quality under drought conditions. *Scientific Papers - Series B, Horticulture*, 201–204.

Serra, I., Strever, A., Myburgh, P. A., and Deloire, A. (2014). Review: the interaction between rootstocks and cultivars (Vitis vinifera L.) to enhance drought tolerance in grapevine. *Australian Journal of Grape and Wine Research* 20, 1–14. doi:10.1111/ajgw.12054.

Simonneau, T., Lebon, E., Coupel-Ledru, A., Marguerit, E., Rossdeutsch, L., and Ollat, N. (2017). Adapting plant material to face water stress in vineyards: which physiological targets for an optimal control of plant water status? *1* 51, 167–179. doi:10.20870/oeno-one.2017.51.2.1870.

Stoll, M., Bischoff-Schaefer, M., Lafontaine, M., Tittmann, S., and Henschke, J. (2013). IMPACT OF VARIOUS LEAF AREA MODIFICATIONS ON BERRY MATURATION IN VITIS VINIFERA L. “RIESLING.” *Acta Hortic.*, 293–299. doi:10.17660/ActaHortic.2013.978.34.

Tissot, C., Neethling, E., Rouan, M., Barbeau, G., Quenol, H., and Le Coq, C. (2017). Modeling Environmental Impacts on Viticultural Ecosystems: A First Case Study in a Regulated Wine Producing Area. *Int. J. Agric. Environ. Inf. Syst.* 8, 1–20. doi:10.4018/IJAEIS.2017070101.

Tomás, M., Medrano, H., Escalona, J. M., Martorell, S., Pou, A., Ribas-Carbó, M., et al. (2014). Variability of water use efficiency in grapevines. *Environmental and Experimental Botany* 103, 148–157. doi:10.1016/j.envexpbot.2013.09.003.

Tomaz, A., Pacheco, C. A., and Coleto Martinez, J. M. (2017). Influence of cover cropping on water uptake dynamics in an irrigated Mediterranean vineyard: Cover Cropping and Water Uptake Dynamics. *Irrig. and Drain.* 66, 387–395. doi:10.1002/ird.2115.

Torres, N., Goicoechea, N., and Carmen Antolín, M. (2018a). Influence of irrigation strategy and mycorrhizal inoculation on fruit quality in different clones of Tempranillo grown under elevated temperatures. *Agricultural Water Management* 202, 285–298. doi:10.1016/j.agwat.2017.12.004.

Torres, N., Goicoechea, N., Zamarreño, A. M., and Carmen Antolín, M. (2018b). Mycorrhizal symbiosis affects ABA metabolism during berry ripening in Vitis vinifera L. cv. Tempranillo grown under climate change scenarios. *Plant Science* 274, 383–393. doi:10.1016/j.plantsci.2018.06.009.

Torres, N., Hilbert, G., Luquin, J., Goicoechea, N., and Antolín, M. C. (2017). Flavonoid and amino acid profiling on Vitis vinifera L. cv Tempranillo subjected to deficit irrigation under elevated temperatures. *Journal of Food Composition and Analysis* 62, 51–62. doi:10.1016/j.jfca.2017.05.001.

Trigo-Córdoba, E., Bouzas-Cid, Y., Orriols-Fernández, I., and Mirás-Avalos, J. M. (2015). Effects of deficit irrigation on the performance of grapevine (Vitis vinifera L.) cv. ‘Godello’ and ‘Treixadura’ in Ribeiro, NW Spain. *Agricultural Water Management* 161, 20–30. doi:10.1016/j.agwat.2015.07.011.

Trouvelot, S., Bonneau, L., Redecker, D., van Tuinen, D., Adrian, M., and Wipf, D. (2015). Arbuscular mycorrhiza symbiosis in viticulture: a review. *Agron. Sustain. Dev.* 35, 1449–1467. doi:10.1007/s13593-015-0329-7.

Valentini, G., Allegro, G., Pastore, C., Colucci, E., and Filippetti, I. (2019). Post-veraison trimming slow down sugar accumulation without modifying phenolic ripening in Sangiovese vines: Post-veraison trimming of Sangiovese vines. *Journal of the Science of Food and Agriculture* 99, 1358–1365. doi:10.1002/jsfa.9311.

van Leeuwen, C., and Darriet, P. (2016). The Impact of Climate Change on Viticulture and Wine Quality. *Journal of Wine Economics* 11, 150–167. doi:10.1017/jwe.2015.21.

Van Leeuwen, C., Pieri, P., Gowdy, M., Ollat, N., and Roby, J.-P. (2019). Reduced density is an environmental friendly and cost effective solution to increase resilence to drought in vineyards in a contexte of climate change. *OENO One* 53, 129–146. doi:10.20870/oeno-one.2019.53.2.2420.

Vaz, M., Coelho, R., Rato, A., Samara-Lima, R., Silva, L. L., Campostrini, E., et al. (2016). Adaptive strategies of two Mediterranean grapevines varieties (Aragonez syn. Tempranillo and Trincadeira) face drought: physiological and structural responses. *Theor. Exp. Plant Physiol.* 28, 205–220. doi:10.1007/s40626-016-0074-6.

Wenter, A., Zanotelli, D., Montagnani, L., Tagliavini, M., and Andreotti, C. (2018). Effect of different timings and intensities of water stress on yield and berry composition of grapevine (cv. Sauvignon blanc) in a mountain environment. *Scientia Horticulturae* 236, 137–145. doi:10.1016/j.scienta.2018.03.037.

Wolkovich, E. M., Burge, D. O., Walker, M. A., and Nicholas, K. A. (2017). Phenological diversity provides opportunities for climate change adaptation in winegrapes. *Journal of Ecology* 105, 905–912. doi:10.1111/1365-2745.12786.

Zhang, L., Marguerit, E., Rossdeutsch, L., Ollat, N., and Gambetta, G. A. (2016). The influence of grapevine rootstocks on scion growth and drought resistance. *Theor. Exp. Plant Physiol.* 28, 143–157. doi:10.1007/s40626-016-0070-x.

Zhu, X., Moriondo, M., van Ierland, E. C., Trombi, G., and Bindi, M. (2016). A model-based assessment of adaptation options for Chianti wine production in Tuscany (Italy) under climate change. *Reg Environ Change* 16, 85–96. doi:10.1007/s10113-014-0622-z.

**Supplementary Figure 1.** Number of results by study and by adaptation indicating a quantitative impact of adaptation on various outputs (FWE: freshwater ecosystem, BC: berry composition, Y: yield, P: phenology, WS: water status), summarized in Figure 5See in the material and method (Section 2) the signification of Negative (N), Neutral and Positive (P) effects.

A) Long-term adaptations


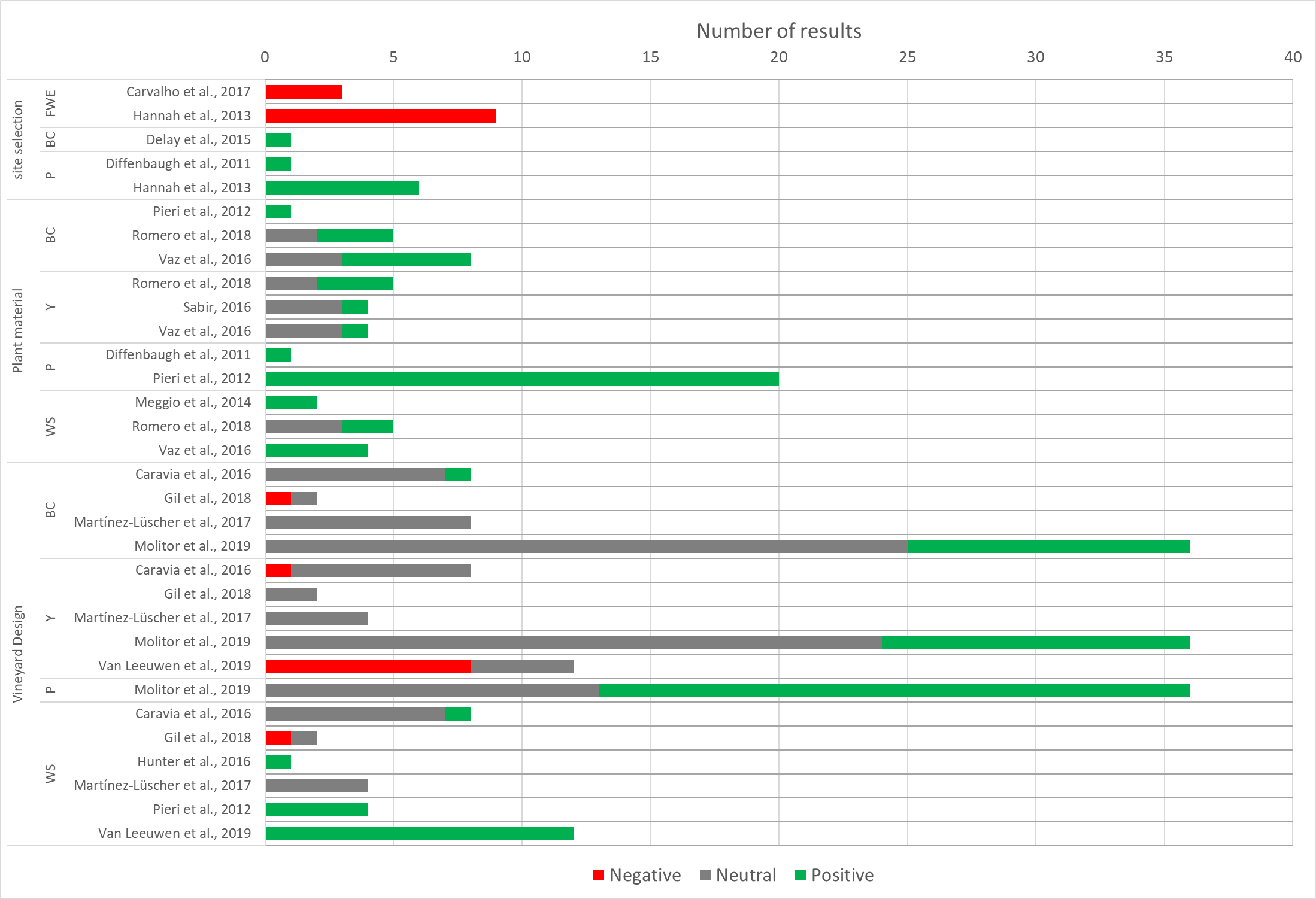


B) Short term adaptations (HM: Harvest and post-harvest management)
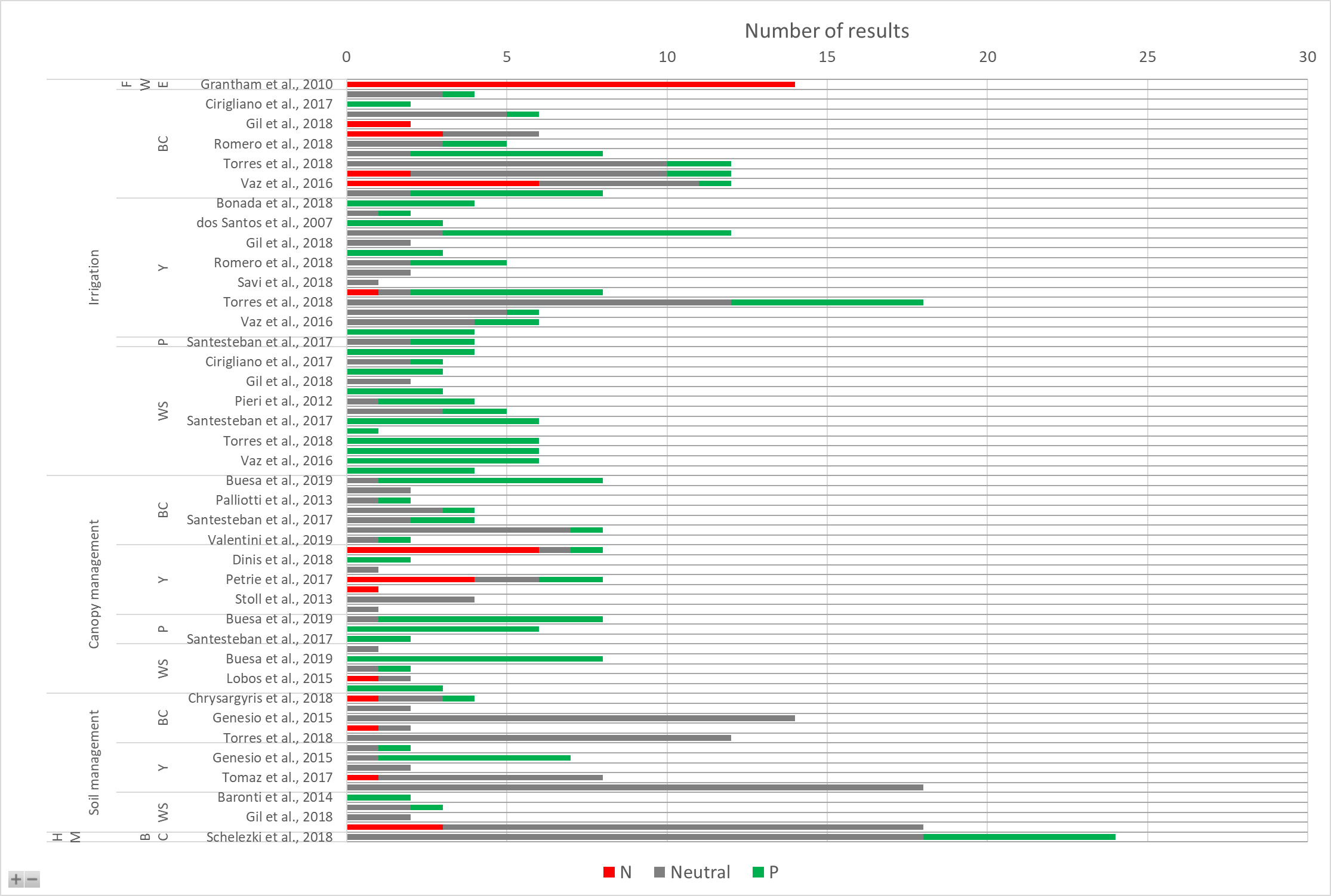


**Supplementary Table 2.** Complete list of indicators of evaluation with associated references (manag = management, select = selection)

|  | | Adaptations |  |  |  |  |  |  |
| --- | --- | --- | --- | --- | --- | --- | --- | --- |
|  | | Irrigation | Plant material | Canopy manag. | Soil manag. | Vineyard design | Farm strategy | Site select. |
| _Plant scale_ | _Gas exchanges*_ | ^[[1]](#endnote-1)^, ^[[2]](#endnote-2)^, ^[[3]](#endnote-3)^, ^[[4]](#endnote-4)^, ^[[5]](#endnote-5)^, ^[[6]](#endnote-6)^ | 4 | ^[[7]](#endnote-7)^,^[[8]](#endnote-8)^ ^[[9]](#endnote-9)^, ^[[10]](#endnote-10)^, ^[[11]](#endnote-11)^, ^[[12]](#endnote-12)^ | 8, 2 | ^[[13]](#endnote-13)^, ^[[14]](#endnote-14)^, 44 |  |  |
|  | _Net photosynthesis_ | 3, 4, 5, ^[[15]](#endnote-15)^ | 4, ^[[16]](#endnote-16)^ | 7, ^[[17]](#endnote-17)^,9, 10, 12 | 15, 8 | 15, 13, 14, 44 |  |  |
|  | _Water Status_ | 1, 3, 4, 5, 6, 15, ^[[18]](#endnote-18)^, ^[[19]](#endnote-19)^, ^[[20]](#endnote-20)^, ^[[21]](#endnote-21)^, ^[[22]](#endnote-22)^, 16, 32 | 4, 16, 21, ^[[23]](#endnote-23)^ | 7, 17, ^[[24]](#endnote-24)^,9, 10, 12 | 15, 8, 20, 22, ^[[25]](#endnote-25)^ | 15, 13, 14, 21 |  |  |
|  | _Genetic_ |  | ^[[26]](#endnote-26)^, ^[[27]](#endnote-27)^, ^[[28]](#endnote-28)^, ^[[29]](#endnote-29)^ |  |  |  |  |  |
|  | _Microclimate_ | 18 |  | 10, 11 |  | 14, 44 |  |  |
|  | _Plant growth_ | 1, 18, 39, 4, 5, 6 | 23, 4, 39 |  | 34 |  |  |  |
|  | _Root dynamics_ | 4 | 4 |  | ^[[30]](#endnote-30)^, 25 |  |  |  |
|  | _Carbon reserve_ |  |  | 12 , ^[[31]](#endnote-31)^ |  |  |  |  |
| _Field scale_ | _Phenology / Suitability_ | 2, ^[[32]](#endnote-32)^ | ^[[33]](#endnote-33)^, 21, 36 | 17, 32, ^[[34]](#endnote-34)^ | 22 | ^[[35]](#endnote-35)^ |  | ^[[36]](#endnote-36)^, ^[[37]](#endnote-37)^ |
|  | _Yield_ | 1, 2, 3, 4, 5, 6, 15, 16, 18, 19, 20, 22, 25, 32, ^[[38]](#endnote-38)^ ^[[39]](#endnote-39)^ | 4, 16, 39 | 10, 12, 17, 31, 32, 34, 43 | 2, 15, 25, 22, ^[[40]](#endnote-40)^ | 15, 35, ^[[41]](#endnote-41)^ |  |  |
|  | _Berry / Wine composition_ | 2, 20, 18, 15, 16, 32, 3, 4, 22, 5, 6 | 4, 7, 21, ^[[42]](#endnote-42)^ | 17, 32, 34, 31, ^[[43]](#endnote-43)^, 12, 7 | 2, 20, 40, 15, 22 | 13, 15, 35, ^[[44]](#endnote-44)^ | ^[[45]](#endnote-45)^ | ^[[46]](#endnote-46)^ |
|  | _Soil properties**_ | 3, 4, 5 | 4 |  | 30, 25 |  |  |  |
|  | _Soil biological properties_ | 22 |  |  | 30, 22 |  |  |  |
|  | _Water use requirement_ | ^[[47]](#endnote-47)^, 5, 38 |  |  |  |  |  |  |
|  | _Run off_ |  |  |  |  |  |  | ^[[48]](#endnote-48)^ |
| _Farm scale_ | _Gross income_ | 3, ^[[49]](#endnote-49)^, ^[[50]](#endnote-50)^, ^[[51]](#endnote-51)^ | 50, 51, ^[[52]](#endnote-52)^ | 50 |  | 50 | 50, 51, 52 | 50 |
|  | _Cost_ | 49, 50, 51 | 50 , 51, 52 |  |  | 50 | 50, 51, 52 | 50 |
|  | _Net revenue_ | 5, 49, 50, 51 | 50, 51, 52 | 50 |  | 50, 41 | 50, 51, 52 | 50, 41 |
|  | _Labour_ |  |  |  |  |  |  | 46 |
|  | _Vulnerability_ | ^[[53]](#endnote-53)^, ^[[54]](#endnote-54)^, ^[[55]](#endnote-55)^ | 53 | 53 | 53 | 53 | 53, ^[[56]](#endnote-56)^ | 53 ,54 |
| _Regional scale_ | _Freshwater ecosystem_ | ^[[57]](#endnote-57)^ |  |  |  |  |  | 37, ^[[58]](#endnote-58)^ |
|  | _Erosion_ |  |  |  |  |  |  | 48, 58 |
|  | _Pollution (N, salinity)_ | 47 |  |  |  |  |  | 58 |
|  | _Economic income_ |  | ^[[59]](#endnote-59)^ |  |  |  |  | 59 |

*A_CO2_, g_s_, WUEi, transpiration, ** soil water content, CEC, P, N, pH, particle size, Corg

1. Bonada et al., 2018 [↑](#endnote-ref-1)
2. Chrysargyris et al., 2018 [↑](#endnote-ref-2)
3. Mirás-Avalos et al., 2016 [↑](#endnote-ref-3)
4. Romero et al., 2018 [↑](#endnote-ref-4)
5. Trigo-Córdoba et al., 2015 [↑](#endnote-ref-5)
6. Wenter et al., 2018 [↑](#endnote-ref-6)
7. Lobos et al., 2015 [↑](#endnote-ref-7)
8. Baronti et al., 2014 [↑](#endnote-ref-8)
9. Attia et al., 2014 [↑](#endnote-ref-9)
10. Dinis et al., 2018 [↑](#endnote-ref-10)
11. Fahey and Rogiers, 2019 [↑](#endnote-ref-11)
12. Palliotti et al., 2013 [↑](#endnote-ref-12)
13. Caravia et al., 2016 [↑](#endnote-ref-13)
14. Hunter et al., 2016 [↑](#endnote-ref-14)
15. Gil et al., 2018 [↑](#endnote-ref-15)
16. Vaz et al., 2016 [↑](#endnote-ref-16)
17. Buesa et al., 2019 [↑](#endnote-ref-17)
18. dos Santos et al., 2007 [↑](#endnote-ref-18)
19. Savi et al., 2018 [↑](#endnote-ref-19)
20. Cirigliano et al., 2017 [↑](#endnote-ref-20)
21. Pieri et al., 2012 [↑](#endnote-ref-21)
22. Torres et al., 2018 [↑](#endnote-ref-22)
23. Meggio et al., 2014 [↑](#endnote-ref-23)
24. de C. Teixeira et al., 2014 [↑](#endnote-ref-24)
25. Tomaz et al., 2017 [↑](#endnote-ref-25)
26. Carvalho et al., 2017 [↑](#endnote-ref-26)
27. Duchene et al., 2012 [↑](#endnote-ref-27)
28. Malacarne et al., 2018 [↑](#endnote-ref-28)
29. Milla‐Tapia et al., 2013 [↑](#endnote-ref-29)
30. Amendola et al., 2017 [↑](#endnote-ref-30)
31. Valentini et al., 2019 [↑](#endnote-ref-31)
32. Santesteban et al., 2017 [↑](#endnote-ref-32)
33. Wolkovich et al., 2017 [↑](#endnote-ref-33)
34. Petrie et al., 2017 [↑](#endnote-ref-34)
35. Molitor et al., 2019 [↑](#endnote-ref-35)
36. Diffenbaugh et al., 2011 [↑](#endnote-ref-36)
37. Hannah et al., 2013 [↑](#endnote-ref-37)
38. Fraga et al., 2018 [↑](#endnote-ref-38)
39. Sabir, 2016 [↑](#endnote-ref-39)
40. Genesio et al., 2015 [↑](#endnote-ref-40)
41. Van Leeuwen et al., 2019 [↑](#endnote-ref-41)
42. Berdeja et al., 2015 [↑](#endnote-ref-42)
43. Stoll et al., 2013 [↑](#endnote-ref-43)
44. Martínez-Lüscher et al., 2017 [↑](#endnote-ref-44)
45. Schelezki et al., 2018 [↑](#endnote-ref-45)
46. Delay et al., 2015 [↑](#endnote-ref-46)
47. Phogat et al., 2018 [↑](#endnote-ref-47)
48. Concepción Ramos, 2016 [↑](#endnote-ref-48)
49. Aparicio et al., 2019 [↑](#endnote-ref-49)
50. Galbreath et al., 2016 [↑](#endnote-ref-50)
51. Sacchelli et al., 2016 [↑](#endnote-ref-51)
52. Georgopoulou et al., 2017 [↑](#endnote-ref-52)
53. Nicholas and Durham, 2012; Lereboullet et al., 2013; Neethling et al., 2017 [↑](#endnote-ref-53)
54. Montaña et al., 2016 [↑](#endnote-ref-54)
55. Ronco et al., 2017 [↑](#endnote-ref-55)
56. Pickering et al., 2014 [↑](#endnote-ref-56)
57. Grantham et al., 2010 [↑](#endnote-ref-57)
58. Carvalho-Santos et al., 2016 [↑](#endnote-ref-58)
59. Zhu et al., 2016 [↑](#endnote-ref-59)
